# Enhanced Recyclability of Thermoplastic Elastomer Toughened Polyamide 6 via Tri- and Multi-epoxy-Terminated POSS Hybrid Additives

**DOI:** 10.1021/acsomega.4c07547

**Published:** 2024-10-30

**Authors:** Rumeysa Yıldırım, Olcay Mert, Güralp Özkoç, Mehmet Kodal

**Affiliations:** † Chemical Engineering Department, Kocaeli University, 41001 Kocaeli, Türkiye; ‡ Polymer Science and Technology Graduate Programme, Kocaeli University, 41001 Kocaeli, Türkiye; § Department of Chemistry, Kocaeli University, 41001 Kocaeli, Türkiye; ∥ Nanotechnology Research and Application Center SUNUM, Sabanci University, 34956 İstanbul, Türkiye; ⊥ Department of Chemistry, İstinye University, 34010 İstanbul, Türkiye; # Xplore Instruments B.V., 6135 KT Sittard, The Netherlands

## Abstract

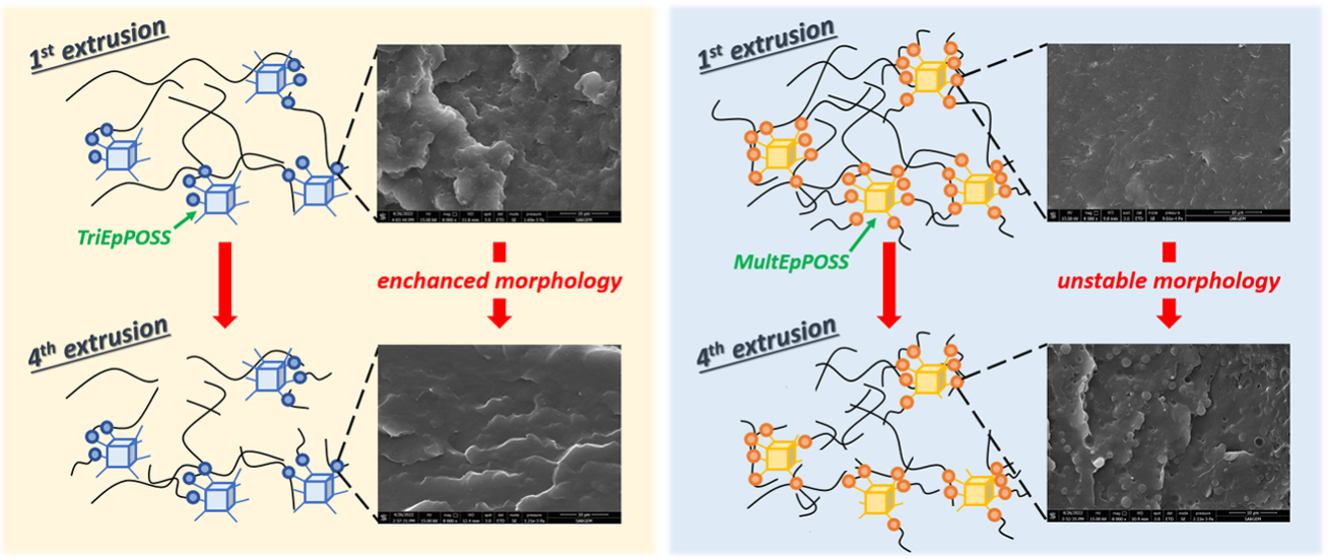

Although widely used polymers, such as polyethylene (PE) and polypropylene (PP), are easily recyclable, complex engineering polymer blends used in the automotive sector involve recycling challenges at the end of life. This study explores the alterations in the properties of compatibilized PA6/TPE blends under multiple thermomechanical recycling conditions, especially for the automotive industry in underhood applications. The compatibilization of PA6/TPE blends was achieved through the incorporation of polyhedral oligomeric silsesquioxane (POSS) nanoparticles, specifically utilizing variants with three and multiple epoxide functional groups (TriEpPOSS and MultEpPOSS, respectively) in their cage structures. Following each recycling step, the blends were pelletized and injection-molded to characterize their morphological, rheological, mechanical, and thermomechanical properties. The findings revealed that the addition of TriEpPOSS significantly improved all properties of PA6/TPE blends throughout successive extrusion cycles. For instance, significant enhancements in Izod impact strength were achieved through the incorporation of TriEpPOSS into the PA6/TPE blend across multiple extrusion cycles. Notably, the 80PA6/20TPE blend with 1 wt % TriEpPOSS demonstrated a remarkable 685% increase in Izod impact strength compared to the same blend without TriEpPOSS following the fourth extrusion cycle. Conversely, the incorporation of MultEpPOSS slightly reduced the rheological and mechanical properties after each extrusion cycle. Nonetheless, it was observed that all properties, particularly rheological characteristics, were superior in blends compatibilized with MultEpPOSS compared to both TriEpPOSS-compatibilized and noncompatibilized blends, owing to the heightened reactivity of MultEpPOSS toward PA6.

## 1 Introduction

Polyamide 6 (PA6) stands out as a notable engineering thermoplastic within numerous spheres of everyday utility, owing to its remarkable attributes such as commendable strength, stiffness, thermal stability, and resilience against chemical degradation and abrasion.^1^ However, the widespread employment of PA6 is restrained by its inherent notch sensitivity. The most widely used strategy to enhance PA6’s toughness lies in its blending with elastomers.^2^ Nevertheless, the environmental friendliness of such blends is compromised due to their challenging recyclability, thereby raising concerns regarding their sustainability.

In recent years, thermoplastic elastomers (TPEs) have garnered significant attention both within industrial applications and academic discourse. These polymeric materials exhibit a unique combination of properties, resembling elastomers in their physical attributes, thermoplastics in their processing capabilities, and a chemical structure that shares characteristics of both thermoplastics and elastomers. Within the TPE family, polyester-based thermoplastic elastomers stand out due to their composition, comprising rigid polyester segments dispersed within a flexible matrix. Noteworthy characteristics of these materials include exceptional dimensional stability, high impact resistance, notable wear and tear resilience, and impressive chemical resistance.^3^ In addition, one of the most notable features distinguishing TPEs from elastomers is their recyclability. Therefore, they are an environmentally friendly alternative material to improve the disadvantageous properties of PA6.

On the other hand, polymer blends exhibit an inherently unstable phase morphology due to their thermodynamically immiscible nature. This characteristic renders the attainment of desired properties through conventional blending methods. It is imperative to mitigate interfacial tension between the polymer constituents, enhance interfacial adhesion, and establish a stable phase morphology to overcome this limitation and achieve the desired properties. Enhancing the interface between the components in polymer blends is generally achieved through compatibilization techniques. Reactive compatibilization is the most effective method to obtain desired properties due to its superior cost-effectiveness and performance attributes.^4−6^ Reactive compatibilization involves chemical reactions between the functional groups present in the compatibilizer, such as epoxy or carboxylic acid groups, and the constituents of the blend during melt blending processes. Recently, polyhedral oligomeric silsesquioxane (POSS) nanoparticles, an organic/inorganic hybrid nanoparticle, have been frequently used for reactive compatibilization of polymer blends. POSSs are emerging as an alternative to conventional nanoparticles due to their flexible physical and chemical properties and their potential to improve the mechanical and thermal properties of polymers/polymer blends.^7,8^

As is widely acknowledged, the exponential growth in global population necessitates a corresponding escalation in consumption demands. This surge in plastic utilization inevitably engenders an influx of plastic waste. Given the nondegradability of plastic materials, which may persist for extended periods, sometimes spanning hundreds of years, their disposal poses a significant environmental concern.^9^ Consequently, heightened environmental consciousness in recent times has spurred the advent of novel strategies in plastic usage. Among these, plastic recycling stands out as a pivotal approach to mitigating the adverse environmental impact of plastics, with mechanical recycling emerging as the predominant method.^10,11^ Notably, this method not only serves to curtail reliance on petroleum-derived resources but also reduces the energy expenditure associated with polymer synthesis, thus yielding economic benefits.

The polyester-based thermoplastic elastomer used in this study to toughen the PA6 stands out for its exceptional heat resistance compared to the other conventional TPEs. Furthermore, its thermoplastic elastomeric nature offers the distinct advantage of recyclability, surpassing conventional rubbers in this regard. However, the recycling of polymeric materials, subject to increased thermal energy during each recycling cycle, can result in molecular weight reduction or structural alterations due to degradation reactions, notably chain scission reactions, thereby compromising the mechanical and thermal properties of the material. As elucidated previously, realizing desired properties in polymer blends necessitates attaining a uniformly dispersed or stable phase morphology, achievable primarily through compatibilization techniques. The stable phase morphology attained via compatibilization of thermodynamically immiscible polymer blends, facilitated by reactive compatibilizers, enhances the recyclability of polymer blends.^12−14^ This is attributed to the stabilizing role of compatibilizers during the recycling process. For instance, Chikh et al. investigated the mechanical, thermal, rheological, and morphological properties of poly(3-hydroxy butyrate-*co*-3-hydroxyvalerate)/poly(butylene succinate) (PHBV/PBS) blends subjected to repeated recycling, revealing that samples containing compatibilizers exhibited elevated values such as storage modulus and tensile strength compared to those without compatibilizers.^14^ Similarly, La Mantia and Capizzi explored the impact of two distinct compatibilizers on the mechanical properties of PA6/polypropylene (PP) blends following repeated extrusion, observing significantly enhanced elongation at break in samples treated with PP compatibilizers containing acrylic acid. This enhancement was ascribed to increased interfacial interactions facilitated by the presence of compatibilizers.^13^ Furthermore, Kudva et al. assessed the efficacy of styrene/acrylonitrile/maleic anhydride terpolymer (SANMA) and imidized acrylic polymer (IA) as compatibilizers on the mechanical, rheological, and morphological properties of PA6/ABS blends postrepeated reprocessing, noting notably higher melt viscosity values in SANMA-compatible PA6/ABS blends over time compared to blends lacking compatibilizers.^12^

In the initial formulation of polymer blends, the objective is to produce blends with optimized, stable, and reproducible properties. Similarly, during recycling, the focus shifts toward re-establishing these desired characteristics. In the current work, the effect of hybrid POSS nanoparticles as compatibilizers on the mechanical, rheological, and morphological properties of TPE-toughened PA6 blends was investigated during repeated extrusion. For this purpose, the blends with and without POSS nanoparticles having triple and multiple epoxy functional groups were melt mixed, granulated, and then reprocessed four times. It was aimed to recompatibilize the PA6/TPE blends in the presence of TriEpPOSS and MultEpPOSS nanoparticles, which have the potential to react with amine, amide, and carboxylic acid reactive groups of PA6 and/or hydroxyl and carboxylic acid reactive groups of TPE via their functional epoxy groups, during repeated extrusion to restore the original blend’s morphology, fortify the structure against potential damage incurred during the forming process, and ensure robust interphase adhesion in the solid state.

## 2 Materials and Methods

### 2.1 Materials

Polyamide 6 (PA6; trade name: Tecomid NB60 NL) and polyester-based thermoplastic elastomer (TPE; trade name: Arnitel UM551) were obtained from Eurotec, Türkiye, and DSM, The Netherlands, respectively. Triglycidylisobutyl POSS (TriEpPOSS) and glycidyl POSS (MultEpPOSS) were used as compatibilizers and obtained from Hybrid Plastics Company. The POSSs are viscous liquids at ambient temperature. Table 1 shows the chemical structures of the materials used in the study.

**Table 1:**
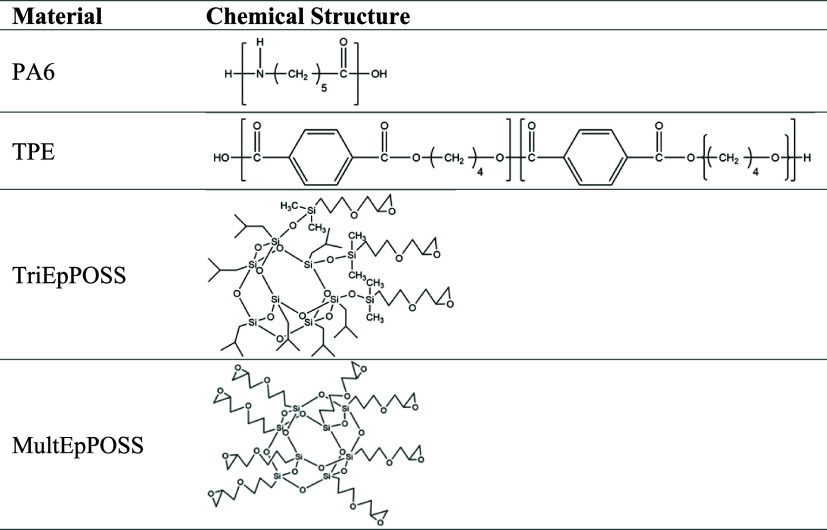
Chemical Structures of Materials

### 2.2 Sample Preparation

**Figure 1:**
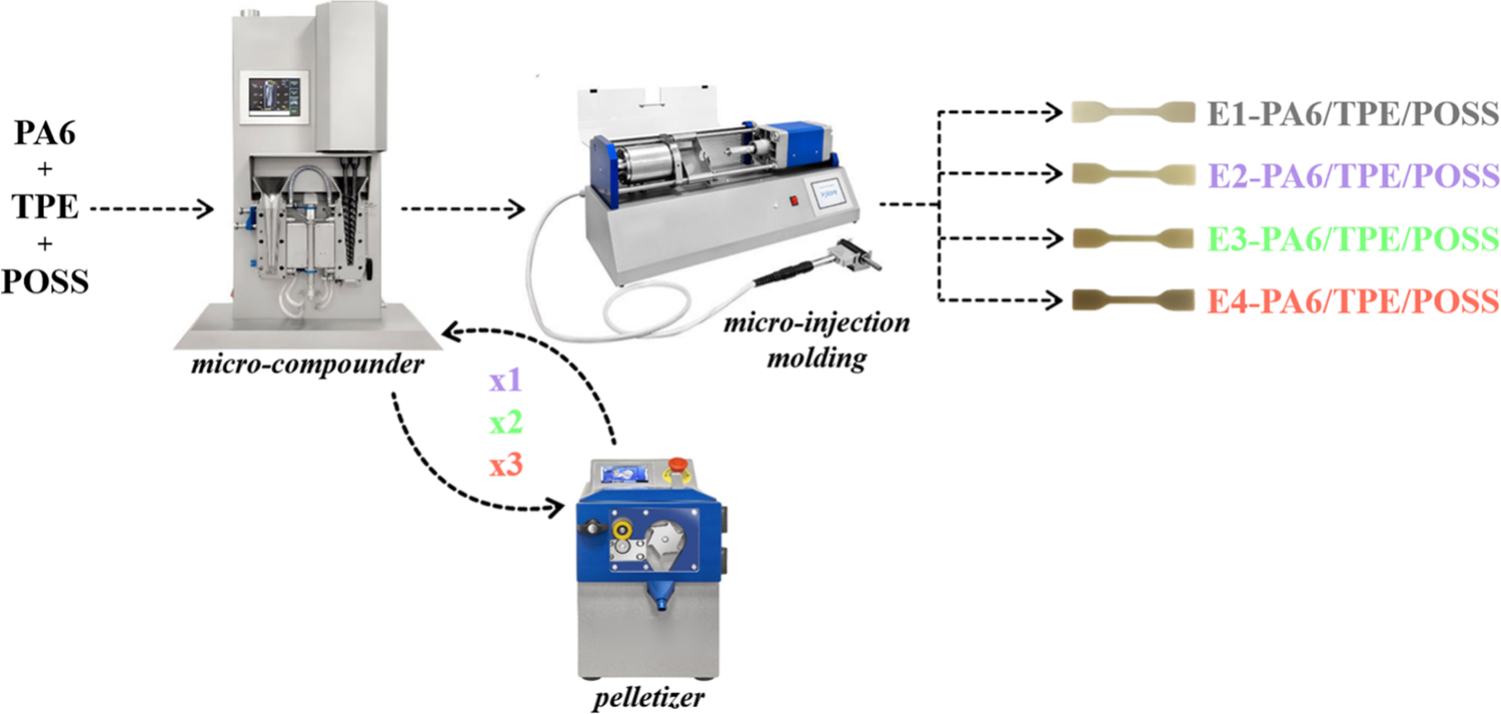
Processing steps of thermomechanical cycles of samples.

Prior to processing, both PA6 and TPE were dried in an oven at 80 °C for 12 h under vacuum. Both types of POSS nanoparticles were incorporated into the PA6/TPE blend at concentrations of 0.5 and 1 wt %. The weight ratio of PA6 to TPE in the blend was 80/20. PA6/TPE and PA6/TPE/POSS blends were compounded for 2 min at 230 °C by the melt blending method in a laboratory scale twin-screw microcompounder (Xplore Instruments 15 cc Microcompounder). The resultant material underwent granulation. Additionally, a series of three consecutive passes were executed under identical processing conditions. Compounded pellets were subsequently subjected to vacuum drying at 80 °C for a duration of 12 h. Following each extrusion cycle, the blends were subjected to molding using a laboratory scale microinjection molding device (Xplore Instruments 12 cc Injection Molder) to fabricate standard test specimens. The injection molding operation was conducted at a pressure of 10 bar, with mold and melt temperatures set at 25 and 230 °C, respectively. Figure 1 illustrates the sequential thermomechanical processing stages. The samples were abbreviated according to the number of extrusion processes. For instance, E2-PA6 denotes the PA6 subjected to two extrusion cycles.

### 2.3 Characterizations

#### 2.3.1 Fourier Transform Infrared (FTIR) Spectroscopy

The possible interactions among PA6, TPE, and POSSs were investigated using a Fourier transform infrared (FTIR) spectroscopy instrument from the PerkinElmer Spectrum 100 model. Samples were positioned on the diamond crystal within the attenuated total reflection (ATR) unit, and spectra were acquired across the wavenumber range of 4000–650 cm^–1^.

#### 2.3.2 Scanning Electron Microscopy (SEM)

The phase morphologies of samples were examined utilizing a QUANTA 400F Field Emission scanning electron microscope (SEM). SEM pictures were acquired from the cryogenically fractured surfaces of the impact specimens. Before analysis, a fine layer of gold was deposited onto the samples to mitigate charging effects. The average particle size of the dispersed phase (*d*_AVG_) was quantified using employing image analysis software, ImageJ.

#### 2.3.3 Rheological Analyses

Rheology analyses were performed on samples of approximately 2 mm thickness using a stress-controlled MCR 102 model Anton Paar rotational rheometer with parallel plate geometry. The heating compartment was purged with nitrogen gas during the analysis. Angular frequency sweep tests were performed in the frequency range from 0.1 to 600 rad/s at 1% shear train and a constant temperature of 230 °C.

#### 2.3.4 Tensile Test

The tensile properties of the samples were evaluated at ambient temperature using an Instron Model 3345 universal tensile testing machine, operating at a crosshead speed of 50 mm/min, in accordance with the ISO 527-5A standard protocol.

#### 2.3.5 Impact Test

The Izod impact strength of the samples was assessed according to the ISO 180 standard, employing an Instron Ceast Resil Impactor. Prior to testing, a V-shaped notch of 2 mm was introduced into each specimen.

#### 2.3.6 Heat Deflection Temperature (HDT) Test

The heat deflection temperatures of samples were determined by heating the samples from 25 to 230 °C at a heating rate of 2 °C/min under 0.46 MPa load.

#### 2.3.7 Differential Scanning Calorimeter (DSC)

The thermal properties of the samples were analyzed by differential scanning calorimetry (DSC) of the Mettler Toledo DSC1 Star model. DSC analyses were carried out in a nitrogen environment at a heating rate of 10 °C/min in the temperature range of 25–250 °C. Percent crystallinity (%*X*_c_) values of the samples were calculated using eq 1
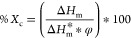
1where Δ*H*_m_ is the enthalpy of melting, Δ*H*_m_* is the enthalpy of melting of 100% crystalline polymer and φ is the mass fraction of polymer components in polymer blends.

#### 2.3.8 Thermogravimetric Analysis (TGA)

The thermal stability of the samples was evaluated using the Mettler Toledo TA Q50 model thermogravimetric analysis (TGA) device. The analyses were carried out in nitrogen atmosphere, in the temperature range of 25–600 °C, at a heating rate of 10 °C/min.

## 3 Results and Discussion

### 3.1 Fourier Transform Infrared (FTIR) Spectroscopy

Samples subjected to extended thermal energy exposure during repeated extrusion processes are susceptible to chemical degradation, which may manifest as thermooxidation and/or photooxidation. Consequently, to enhance comprehension of the potential degradation mechanisms inherent in repeated extrusion, it is imperative to collectively investigate the physical and chemical alterations induced by the resultant degradation products.^15^ The physical alterations observed in the samples subsequent to repeated processing may be delineated as photooxidation, denoting the fragmentation of polymer chains catalyzed by the synergistic action of light and oxygen. This phenomenon invariably precipitates discernible alterations in surface coloration.^16^

**Figure 2:**
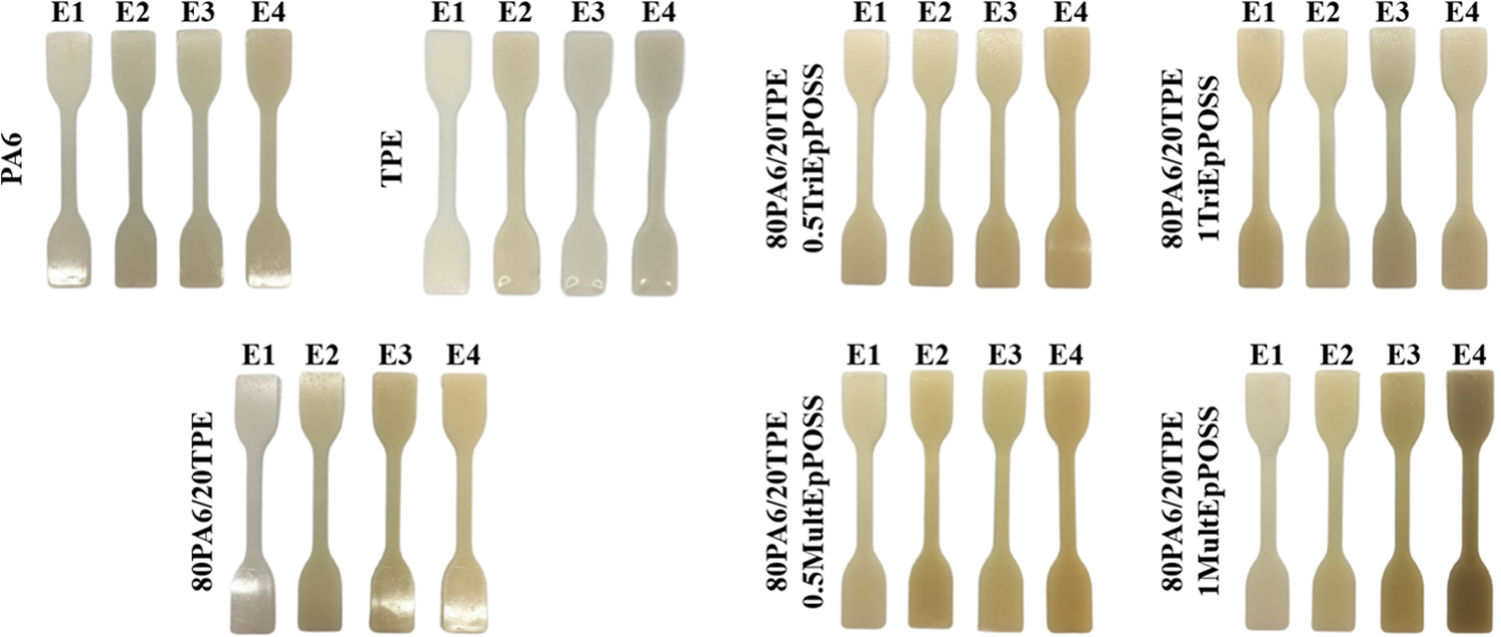
Color changes in PA6, TPE, 80PA6/20TPE, and 80PA6/20TPE/POSS samples as a function of repeated extrusion cycles.

A discernible darkening of sample coloration was noted in each sample with a concomitant rise in the number of extrusion cycles, as illustrated in Figure 2. This observed phenomenon can be attributed to the increased formation of chromophoric entities, notably carbonyl groups, stemming from chain scission events occurring throughout processing. These emergent chromophores significantly contribute to the observed color variation across the samples.^17^ Notably, it is pertinent to highlight the markedly elevated degree of color alteration observed in PA6/TPE blends incorporating MultEpPOSS compared to counterparts devoid of this additive.

Upon examination of the FTIR spectra of PA6, TPE, and 80PA6/20TPE blends subjected to repeated extrusion, a direct correlation emerged between the intensity of the carbonyl peak (–C=O) and the number of extrusion cycles undertaken (Figures S1–S3). This increment in carbonyl peak intensity can be attributed to the occurrence of chain scission phenomena characteristic of polyamide and TPE degradation processes, resulting in the formation of carbonyl functional groups, namely ketones (R–CO–R), aldehydes (R–CO–H), and carboxylic acids (R–CO–OH).^18−21^ The amplification in carbonyl peak intensities observed across samples subjected to repeated extrusion procedures thus delineates the progressive accrual of these carbonyl groups consequent to degradation.^22,23^

In the FTIR spectra of the 80PA6/20TPE blends containing POSS nanoparticles, it was observed that the reactive epoxy group absorption peaks located at 1254, 908, and 838 cm^–1^ for TriEpPOSS nanoparticle and 908, 852, and 836 cm^–1^ for MultEpPOSS nanoparticle were not observed in the blends (Figures S4 and S5). This observation suggests interactions among the reactive amine (−NH_2_), amide (−NHCO−), and carboxylic acid (−COOH) groups of PA6, the reactive carboxylic acid and hydroxy groups of TPE, and easily reacting functional epoxies in the cage of POSSs.^24−26^

**Figure 3:**
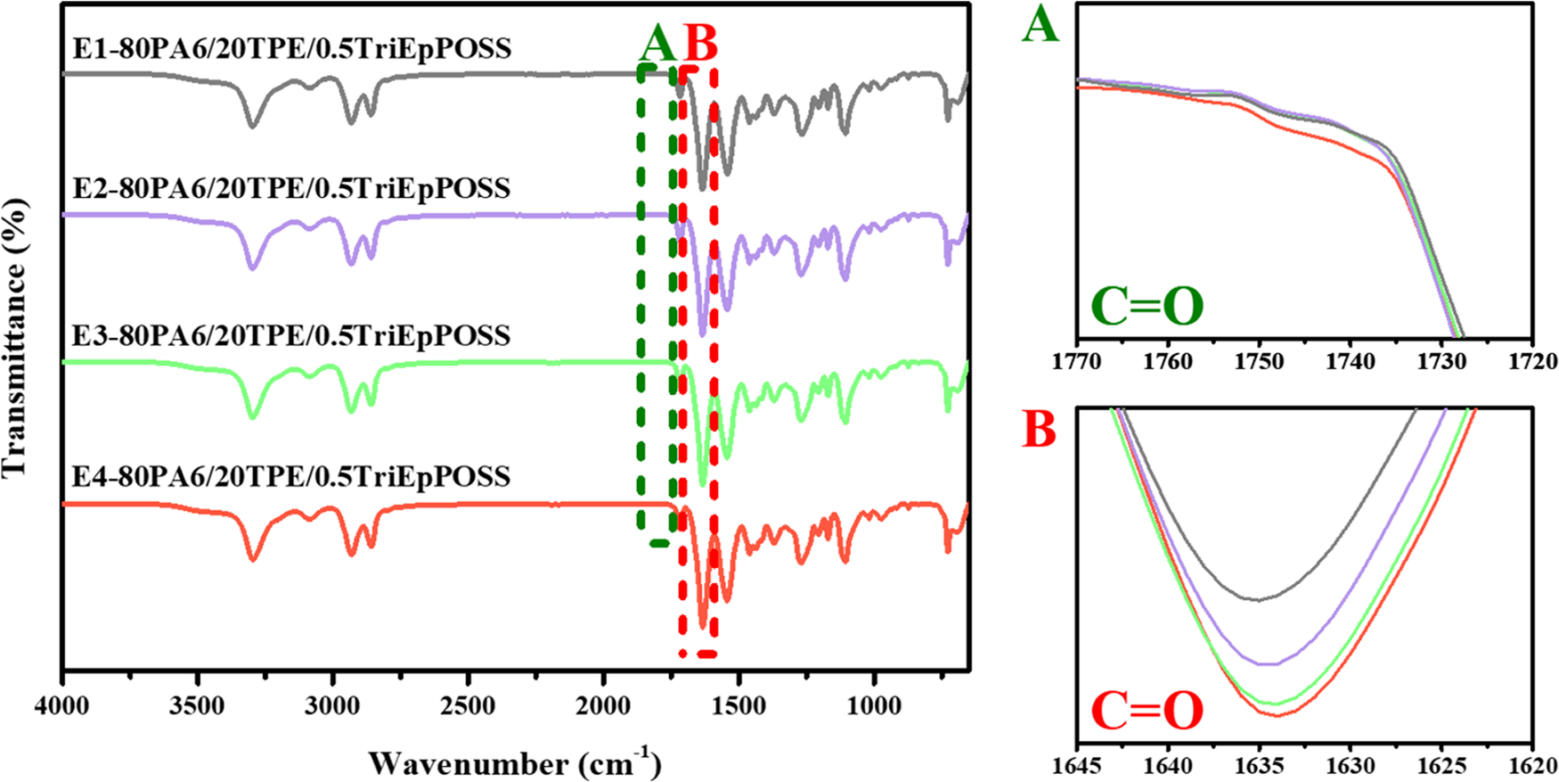
FTIR spectra of 80PA6/20TPE/0.5TriEpPOSS blends after repeated extrusion cycles; (A) 1770–1720 cm^–1^ region, (B) 1645–1620 cm^–1^ region.

**Figure 4:**
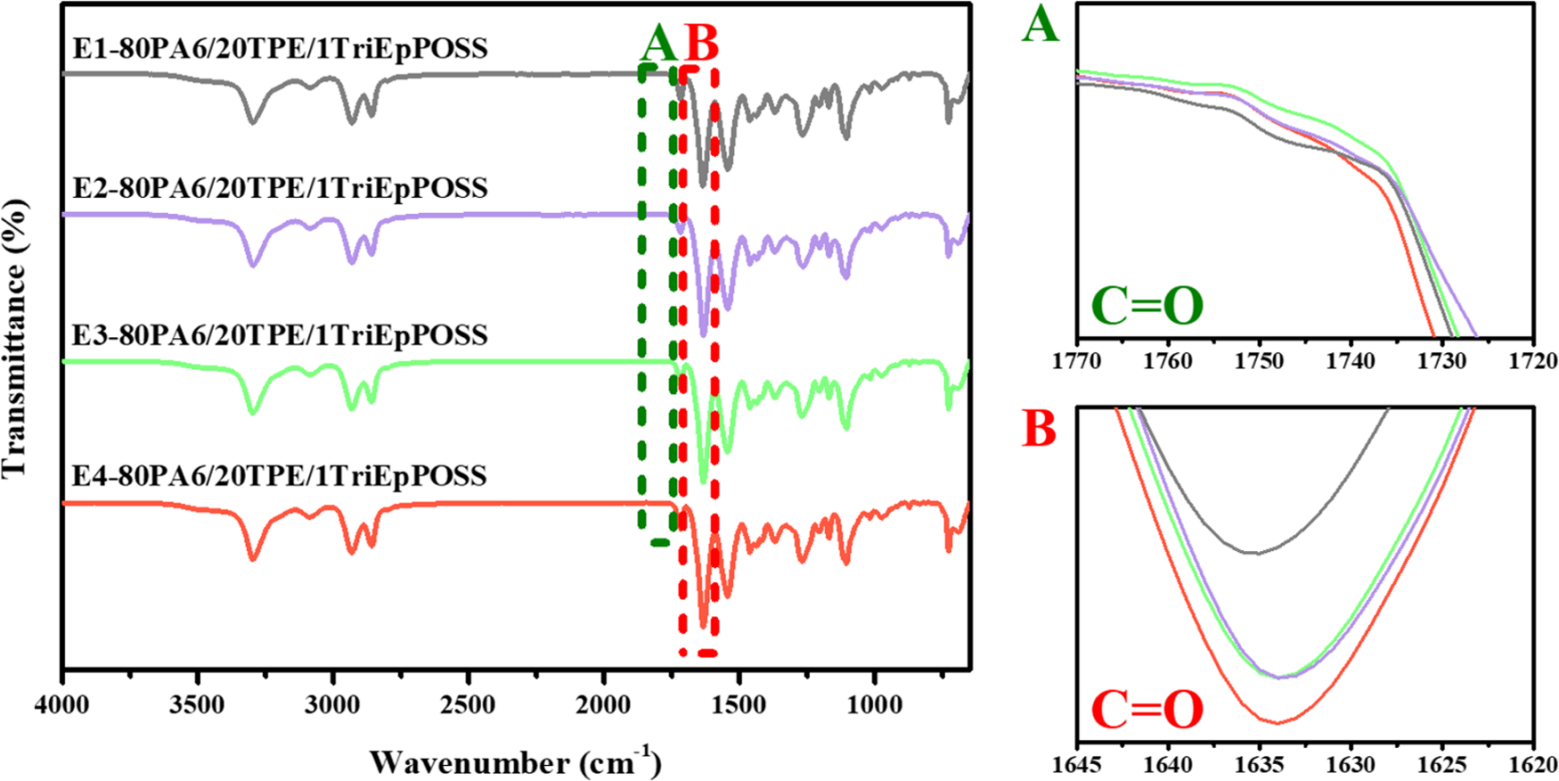
FTIR spectra of 80PA6/20TPE/1TriEpPOSS blends after repeated extrusion cycles; (A) 1770–1720 cm^–1^ region, (B) 1645–1620 cm^–1^ region.

**Figure 5:**
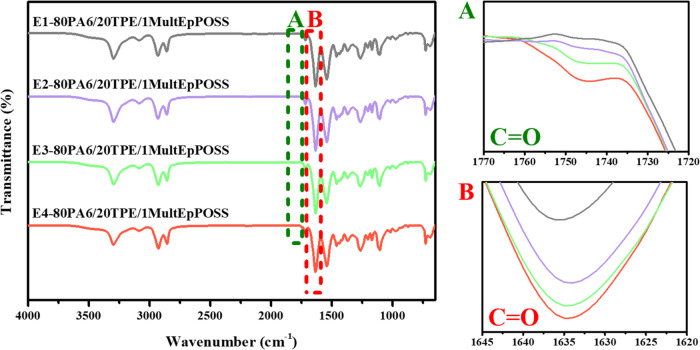
FTIR spectra of 80PA6/20TPE/0.5 MultEpPOSS blends after repeated extrusion cycles; (A) 1770–1720 cm^–1^ region, (B) 1645–1620 cm^–1^ region.

**Figure 6:**
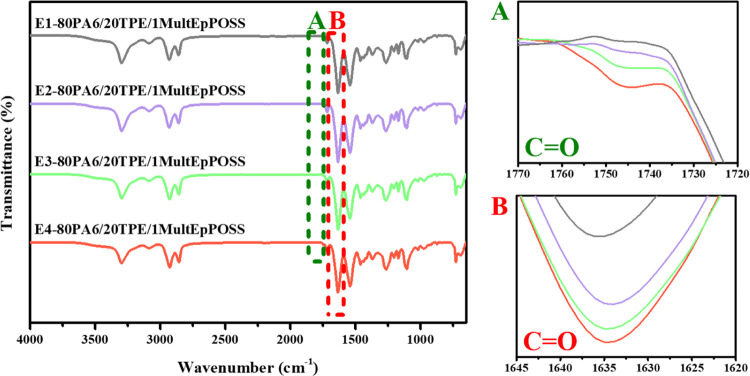
FTIR spectra of 80PA6/20TPE/1MultEpPOSS blends after repeated extrusion cycles; (A) 1770–1720 cm^–1^ region, (B) 1645–1620 cm^–1^ region.

Regardless of the type of POSS and the loading ratio, the formation of carbonyl groups occurred within the 80PA6/20TPE/POSS blends consequent to the degradation reactions catalyzed by repeated extrusion, similar to those observed within the 80PA6/20TPE blends. This phenomenon is evidenced by the heightened intensities of carbonyl peaks in the absorption band of PA6 at 1634 cm^–1^ (Figures 3–6A). Additionally, a distinctive observation emerged in the repeatedly extruded blends containing MultEpPOSS, wherein new peak formations within the range of 1770–1720 cm^–1^ were discerned, particularly within the blends incorporating 1 wt % MultEpPOSS, contrasting with the blends containing TriEpPOSS (Figure 6A).

**Figure 7:**
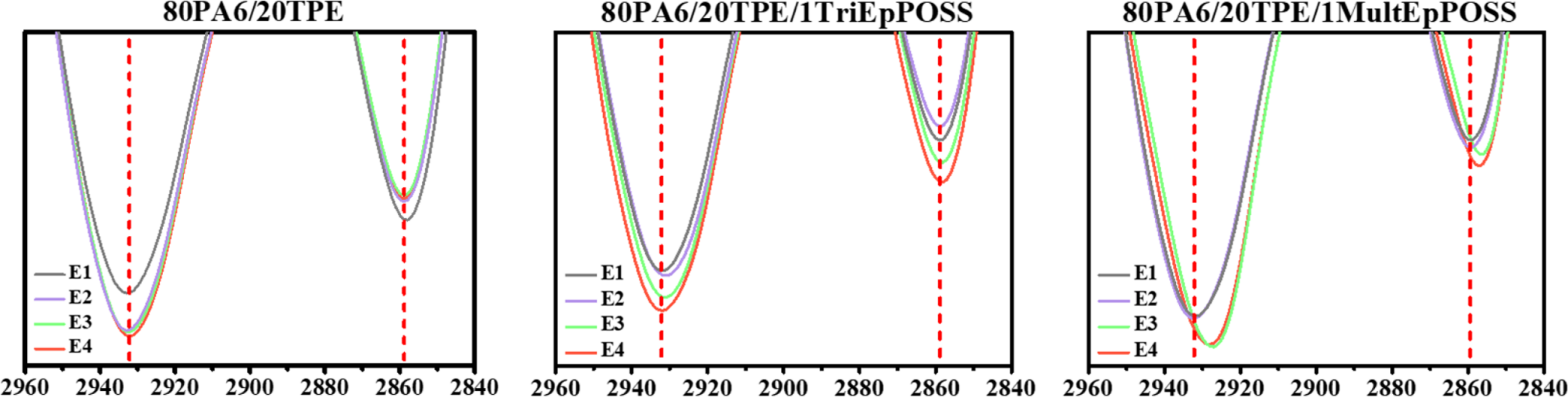
Changes in CH_2_ absorption peak of 80PA6/20TPE, 80PA6/20TPE/TriEpPOSS and 80PA6/20TPE/MultEpPOSS blends after repeated extrusion cycles.

**Scheme 1:**
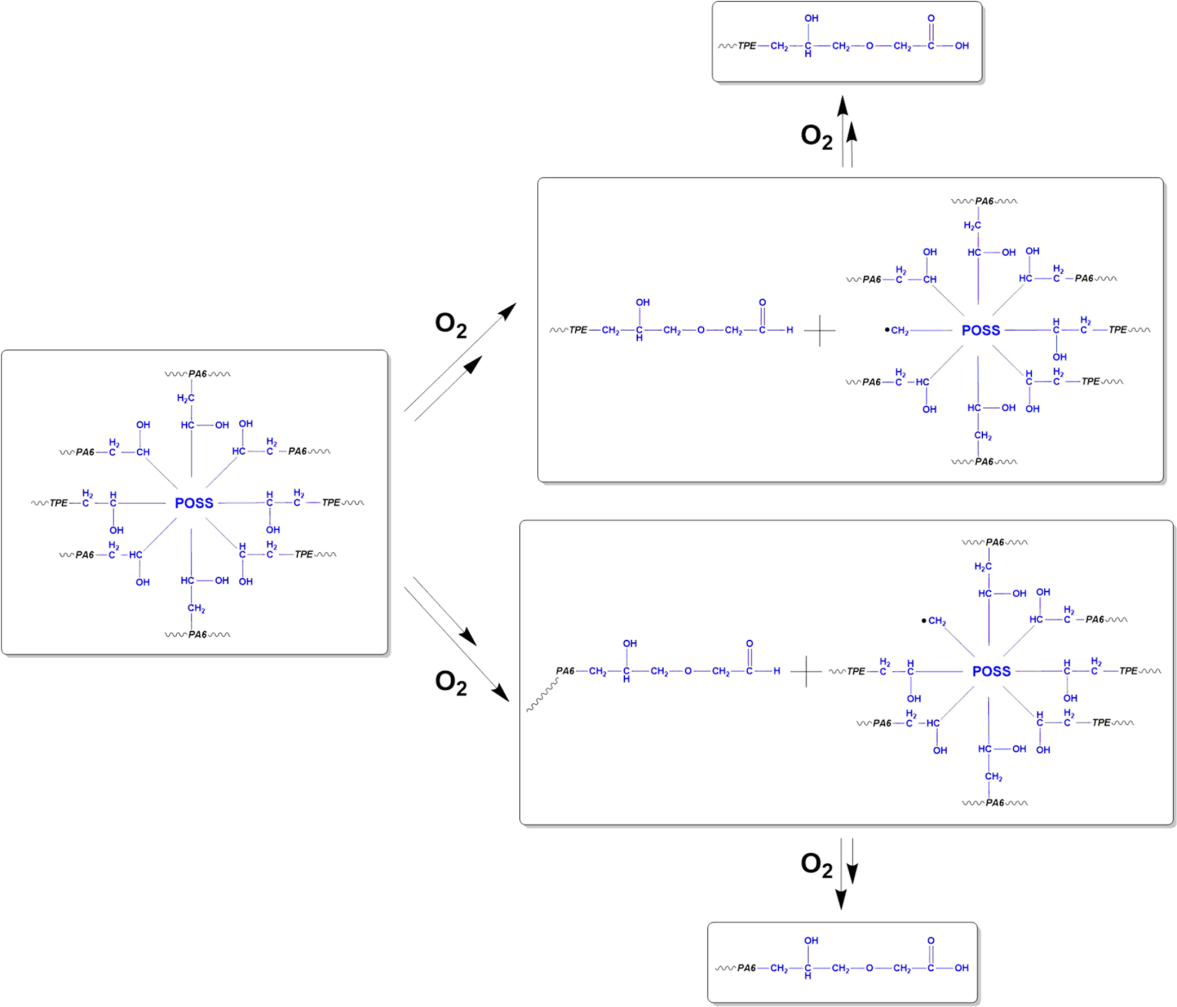
Mechanism of Degradation of MultEpPOSS in the 80PA6/20TPE Blend during the Repeated Extrusion Process

In order to elucidate the origins of the carbonyl peak within the 1770–1720 cm^–1^ range, an examination of the variations in the methylene (−CH_2_) bands of 80PA6/20TPE blends with and without TriEpPOSS and MultEpPOSS compatibilizers was conducted. As depicted in Figure 7, unlike the 80PA6/20TPE and 80PA6/20TPE/TriEpPOSS blends, the peaks in the region of 2960–2840 cm^–1^ corresponding to methylene groups exhibited rightward shifts in the repeatedly processed blends containing MultEpPOSS, correlating with an increase in the number of repeated processing cycles. As illustrated in Scheme 1, these shifts in the methylene bands coincide temporally with the emergence of new carbonyl peaks within the 1770–1720 cm^–1^ region, indicative of chain scission reactions and consequent formation of carbonyl groups within the MultEpPOSS nanoparticle during repetitive processing.^21,27^ Consequently, MultEpPOSS exhibits susceptibility to thermooxidative degradation.^28^ Therefore, the formation of carbonyl groups stemming from MultEpPOSS degradation during repeated extrusion processes instigates the appearance of novel peaks within the 1770–1720 cm^–1^ region (Figure 6A). This phenomenon is posited as the underlying cause of the pronounced color alteration observed in 80PA6/20TPE/MultEpPOSS blends subjected to repetitive processing as shown in Figure 2.

### 3.2 Scanning Electron Microscopy (SEM)

SEM analyses were conducted to ascertain the influence of repeated extrusion processes on the phase stability of the samples. For this purpose, the fracture surfaces of samples subjected to single and multiple extrusions were meticulously examined through cryogenic fracturing of impact test specimens.

**Figure 8:**
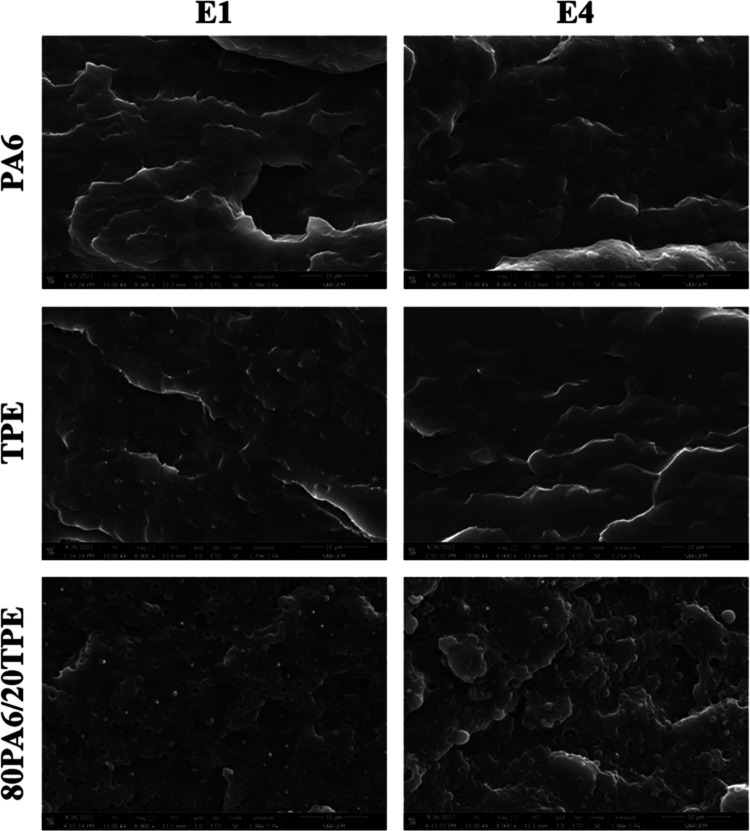
SEM images of PA6, TPE, and the 80PA6/20TPE blend after the first and fourth reprocessing cycles.

As seen in Figure 8, after the first extrusion step, pure PA6 and pure TPE revealed a consistently smooth surface morphology. Subsequent repeated extrusion yielded no observable alterations in the morphological characteristics of either PA6 or TPE. However, an evident transition to a two-phase morphological architecture was evident in the 80PA6/20TPE blend. The incorporation of 20 wt % TPE into PA6 resulted in the presence of TPE particles within the dispersed phase, characterized by an average particle size (*d*_AVG_) of 1.3 μm (Table 2). This outcome can be attributed to the inherent thermodynamic immiscibility between PA6 and TPE, leading to a weak interfacial interaction. Notably, the *d*_AVG_ value of the dispersed phase escalated with processing cycles of the 80PA6/20TPE blend. This phenomenon arises from the progressive reduction in the molecular weight of both PA6 and TPE, induced by chain scission reactions occurring during prolonged exposure to thermal energy. Consequently, diminishing molecular weights corresponded to lowered melt viscosity values and mitigated shear forces during extrusion, thereby facilitating an increase in the average particle size of the dispersed phase.

**Table 2:** Average Particle Sizes of the Dispersed Phase in the Blends after the First and Fourth Reprocessing Cycles

**sample**	***d***_**AVG**_**(μm)**
E1-80PA6/20TPE	1.3 ± 0.2
E4-80PA6/20TPE	1.4 ± 1.0
E1-80PA6/20TPE/0.5TriEpPOSS	0.8 ± 0.2
E4-80PA6/20TPE/0.5TriEpPOSS	0.3 ± 0.1
E1-80PA6/20TPE/1TriEpPOSS	0.7 ± 0.2
E4-80PA6/20TPE/1TriEpPOSS	0.2 ± 0.1
E1-80PA6/20TPE/0.5MultEpPOSS	0.3 ± 0.1
E4-80PA6/20TPE/0.5MultEpPOSS	0.9 ± 0.1
E1-80PA6/20TPE/1MultEpPOSS	0.2 ± 0.1
E4-80PA6/20TPE/1MultEpPOSS	1.5 ± 0.3

**Figure 9:**
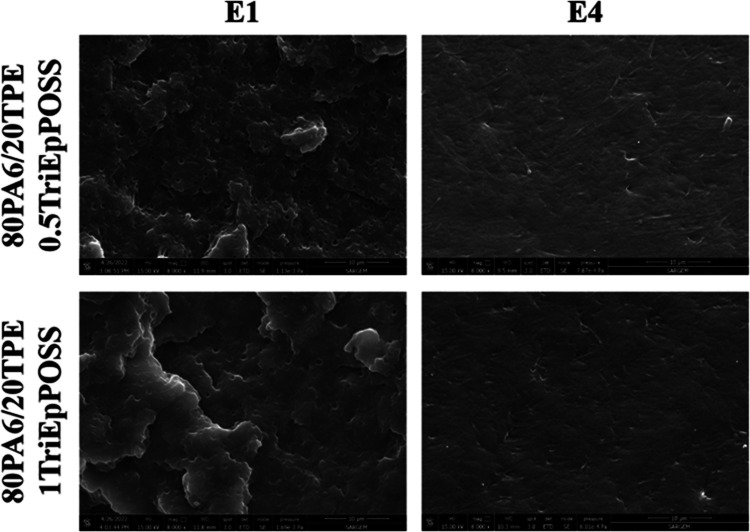
SEM images of the 80PA6/20TPE/TriEpPOSS blend after the first and fourth reprocessing cycles.

It was determined that the average particle size of the dispersed phase decreased upon incorporating TriEpPOSS into the 80PA6/20TPE blend. For instance, in the E1-80PA6/20TPE sample, the average particle size of the dispersed TPE phase, initially measured at 1.3 μm, decreased to 0.8 μm upon the addition of 0.5% TriEpPOSS by weight. The subsequent increase in TriEpPOSS content led to a further reduction in the average particle size of the dispersed TPE phase, reaching 0.7 μm. Notably, a surface morphology predominantly characterized by a single phase was observed. These findings underscored the role of TriEpPOSS as an emulsifier within PA6/TPE blends, enhancing interfacial interactions (Figure 9). Moreover, repeated extrusion of 80PA6/20TPE/TriEpPOSS blends resulted in surface morphologies exhibiting nearly homogeneous single-phase behavior. In light of these outcomes, it is posited that the interfacial interaction between PA6 and TPE is notably enhanced with the incorporation of TriEpPOSS nanoparticles, thereby increasing the particle–matrix interfacial area through a significant reduction in the *d*_AVG_ of the dispersed phase. Additionally, the introduction of TriEpPOSS nanoparticles to PA6/TPE blends was observed to enhance their recyclability properties upon repeated processing, as compatibilizers serve to stabilize the material during recycling processes. Therefore, it can be concluded that TriEpPOSS molecules recompatibilized the interface between the components during the repeated extrusion process. It is imperative to replicate the morphology of the initial blend, fortify the structure against potential damage incurred during the formation process, and ensure robust interfacial adhesion between phases in the solidified state.^29^

**Figure 10:**
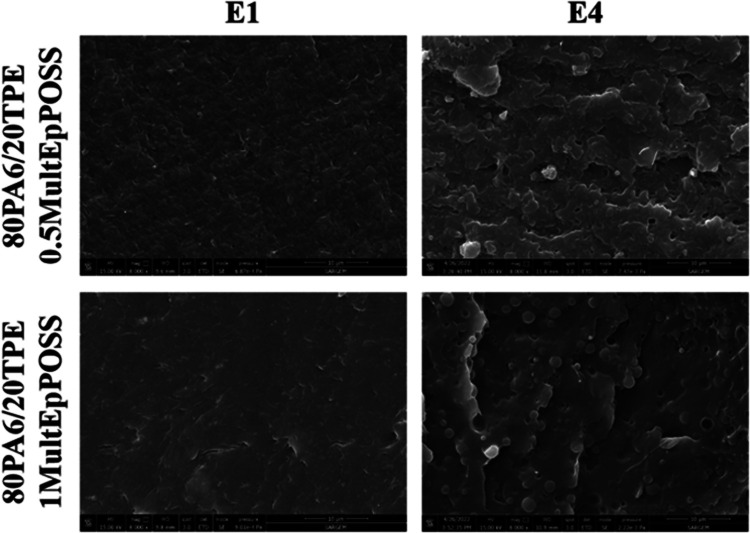
SEM images of 80PA6/20TPE/MultEpPOSS blend after the first and fourth reprocessing cycles.

As expected, incorporating the more reactive MultEpPOSS, which includes multiple epoxy groups, yielded a considerably more stable phase morphology in the E1-80PA6/20TPE blends, as shown in Figure 10. This enhancement arises from the interaction between epoxy functional groups of MultEpPOSS and the end groups of PA6 and/or TPE, as well as with the amide group within the primary backbone of PA6, facilitating the formation of PA6-*g*-POSS-*g*-TPE and/or block copolymers at the interphase. Consequently, the interfacial compatibility among the constituents of the blend is enhanced. Upon comparing 80PA6/20TPE blends containing POSS molecules with those processed once, it has been discerned that MultEpPOSS manifests lower values in the average particle size of the dispersed phase across both loading rates. This outcome is attributable to the heightened reactivity inherent to MultEpPOSS, owing to its increased epoxy group content (Table 2). However, the *d*_AVG_ values of the 80PA6/20TPE/MultEpPOSS blends subjected to four times thermomechanical processing displayed notably larger values compared to the E1-80PA6/20TPE/MultEpPOSS blends. This discrepancy indicates that the phase morphology could not be ameliorated in samples subjected to repeated extrusion processes in the presence of MultEpPOSS, unlike the blends containing TriEpPOSS. This phenomenon can be elucidated by the high reactivity of MultEpPOSS, which tends to undergo complete reaction during the initial extrusion stage and subsequently degrade due to the depletion of epoxy groups available for further reactions upon subsequent processing. Consequently, with an increase in the number of repetitive processes, the prevalence of chain scission reactions ensued, resulting in a reduction in the molecular weight of the samples and consequently rendering the phase morphology more unstable.

### 3.3 Rheological Analyses

The dynamic rheological characteristics of polymers are significantly influenced by variations in their molecular architecture. Consequently, examining rheological properties offers a means to assess the interaction between polymers under specific conditions.^24^ Therefore, rheological analyses were conducted to ascertain the complex viscosity, storage modulus, and loss modulus of the samples generated through repeated extrusion processes.

**Figure 11:**
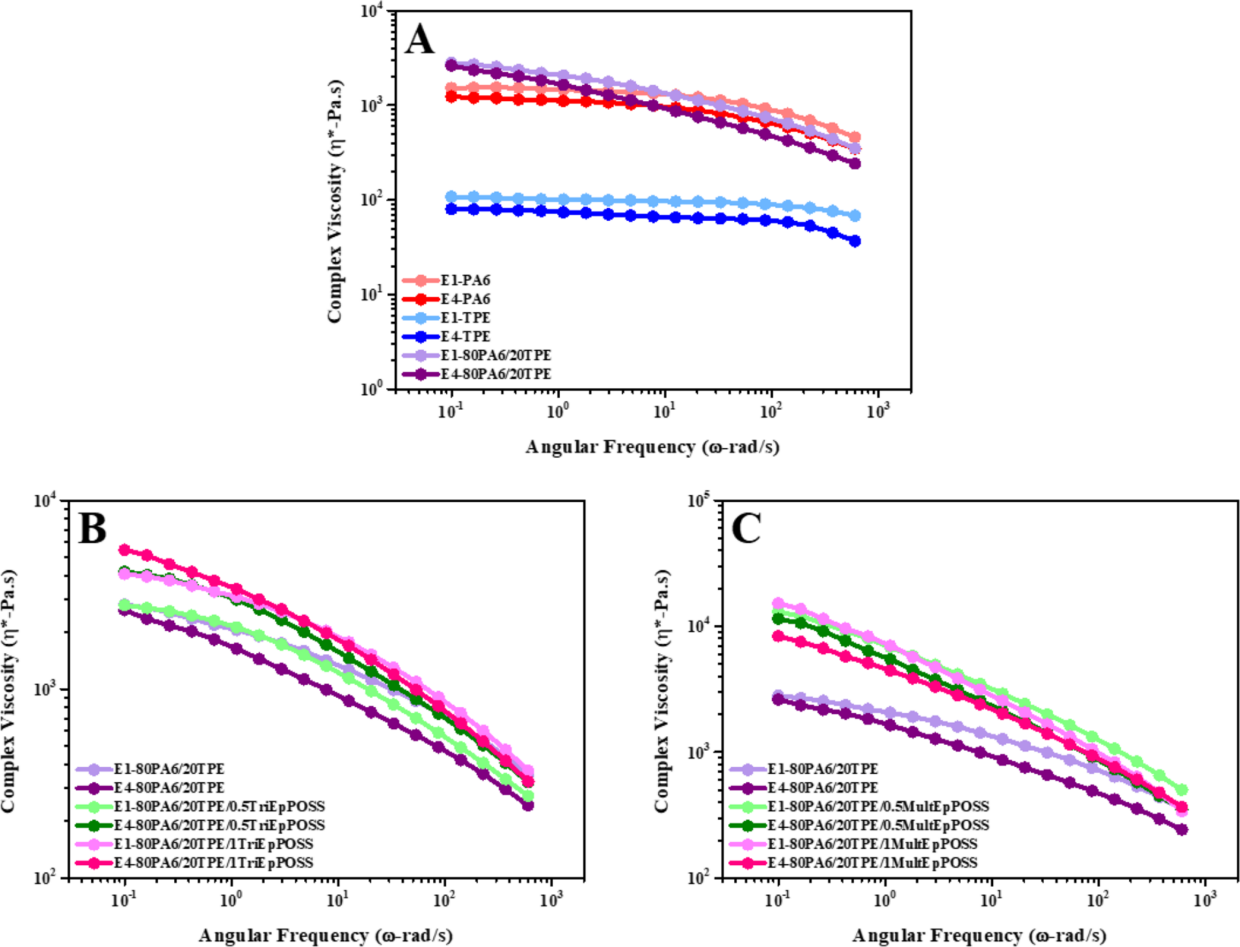
Complex viscosity versus angular frequency of samples after the first and fourth reprocessing cycles; (A) PA6, TPE, and 80PA6/20TPE blend, (B) 80PA6/20TPE and 80PA6/20TPE/TriEpPOSS blends, (C) 80PA6/20TPE and 80PA6/20TPE/MultEpPOSS blends.

A notable decline in the complex viscosity values of pure PA6 was observed as the angular frequency increased (Figure 11A). This reduction was particularly conspicuous at higher frequency values attributable to the shear thinning behavior of PA6.^30^ Furthermore, as previously mentioned, the degradation of PA6 during repeated processing induces chain scission reactions. Consequently, an increment in the number of extrusions led to diminished complex viscosity values across the entire angular frequency range, owing to the presence of lower molecular weight chains stemming from such scission events.^31^ Conversely, pure TPE exhibited an extensive Newtonian plateau region alongside evidence of shear thinning phenomena at elevated angular frequencies.^32^ Similar to pure PA6, pure TPE samples exhibited lower complex viscosity values over the entire angular frequency range with increasing repeated extrusion process.^33^

The incorporation of TPE into pure PA6 yielded increased complex viscosity values compared to pure PA6. This phenomenon is attributed to the formation of hydrogen bonds between the N–H proton of PA6 and the carbonyl functional groups of TPE.^24^ With an increase in the number of extrusion cycles, the complex viscosity values of the blends shifted a downward trend. This phenomenon is corroborated by the findings of FTIR analyses, wherein it was demonstrated that PA6/TPE blends, subjected to prolonged heat treatment due to repeated extrusions, undergo degradation. Consequently, such thermal exposure induces chain scission reactions, culminating in a decline of the complex viscosity values of PA6/TPE blends.^34^

In the E1-80PA6/20TPE blend, the incorporation of POSS led to elevated complex viscosity values, regardless of the POSS type. Moreover, it further increased as the loading ratio of POSS nanoparticles increased in the E1-80PA6/20TPE blend. As an illustration, the complex viscosity value of 2807 Pa·s for the E1-80PA6/20TPE blend at an angular frequency of 0.1 rad/s increased to 4073 and 15,263 Pa·s for blends containing 1 wt % of TriEpPOSS and MultEpPOSS, respectively (Figure 11B,C). This elevation in complex viscosity arises from the interaction between the functional epoxy groups of POSS nanoparticles and reactive groups, including amine, amide, and carboxylic acid of PA6 or hydroxyl groups present within the chain ends of TPE. These interactions facilitate the formation of graft and/or block copolymers within the interfacial region.^24,35^ Furthermore, the greater abundance of reactive epoxy groups in the structure of MultEpPOSS, relative to TriEpPOSS, enhances the propensity for generating higher molecular weight, thereby leading to increased complex viscosity values. Furthermore, the complex viscosities of 80PA6/20TPE/TriEpPOSS blends exhibited frequency-dependent behavior, manifesting shear thinning characteristics indicative of highly branched polymeric structures. This phenomenon serves as further evidence corroborating the formation of graft and/or block copolymers in the presence of TriEpPOSS.

**Figure 12:**
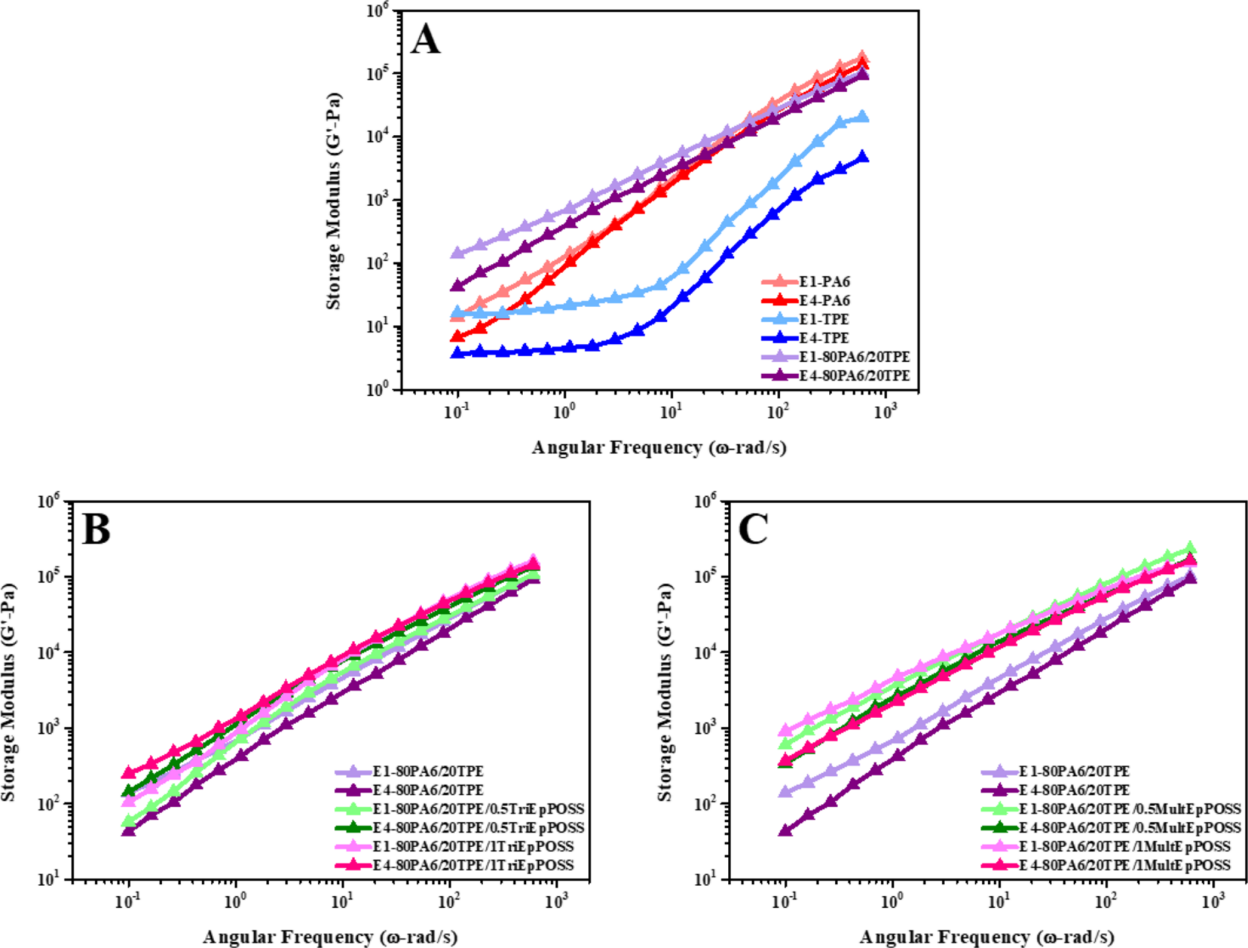
Storage modulus versus angular frequency of samples after the first and fourth reprocessing cycles; (A) PA6, TPE, and 80PA6/20TPE blend, (B) 80PA6/20TPE and 80PA6/20TPE/TriEpPOSS blends, (C) 80PA6/20TPE and 80PA6/20TPE/MultEpPOSS blends.

**Figure 13:**
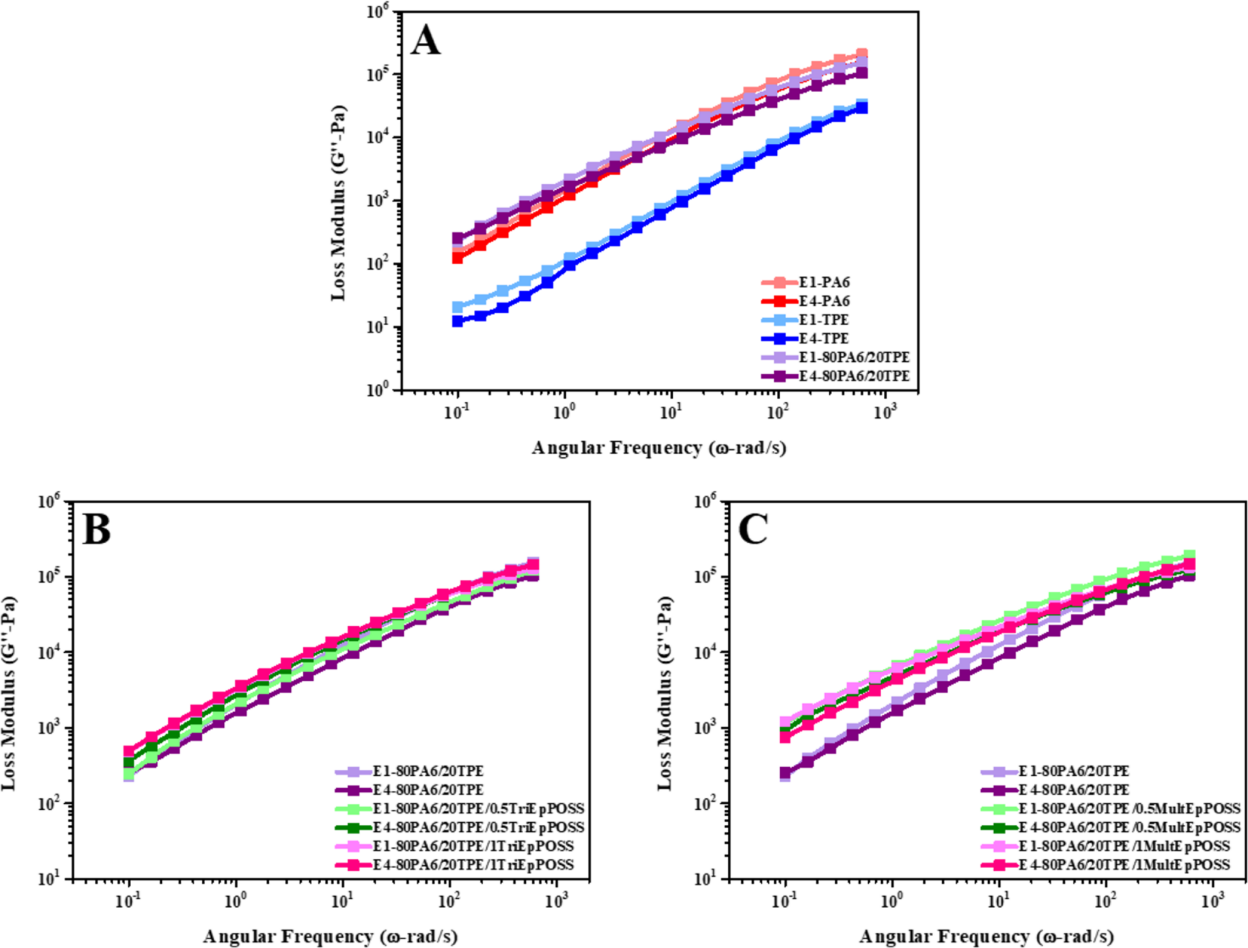
Loss modulus versus angular frequency of samples after the first and fourth reprocessing cycles; (A) PA6, TPE, and 80PA6/20TPE blend, (B) 80PA6/20TPE and 80PA6/20TPE/TriEpPOSS blends, (C) 80PA6/20TPE and 80PA6/20TPE/MultEpPOSS blends.

As illustrated in Figures 12A and 13A, a comprehensive examination of the storage and loss modulus within the E1-PA6 sample reveals consistently higher values in the loss modulus relative to the storage modulus throughout the entire angular frequency range, attributable to the viscous character of PA6.^36^ Similarly, within the E1-TPE sample, a conspicuous dominance of the viscous property is observed by the higher magnitude of the loss modulus compared to the storage modulus. The repeated extrusion processing engenders the formation of short chains due to the reduced molecular weight, thereby resulting in a decrement in both the storage and loss modulus of the samples. In addition, the storage modulus further decreases. This is thought to be because the effect of chain scission is more pronounced since the storage modulus indicates the elastic nature of polymeric materials. Similarly, repeated extrusion cycles of the 80PA6/20TPE blend precipitate a concomitant decline in the storage and loss modulus, consequent to the formation of short chains.

Enhanced storage and loss modulus values were consistently observed upon the incorporation of POSSs into the E1-80PA6/20TPE blend, regardless of the specific type of POSS (Figures 12 and13). This phenomenon finds its rationale in the introduction of TriEpPOSS or MultEpPOSS, which engenders heightened storage and loss modulus values relative to the blend without compatibilizer. This enhancement is attributed to the formation of graft and/or block copolymers at the interfacial region, facilitated by reactions between the epoxy functionalities of POSS nanoparticles and the reactive groups present in PA6 and/or TPE.^37^ Furthermore, it is noteworthy that the increase in storage modulus values outpaces that of the loss modulus upon POSS inclusion. This disparity underscores the pronounced contribution of POSS to the elastic properties of the blends.^38^

**Scheme 2:**
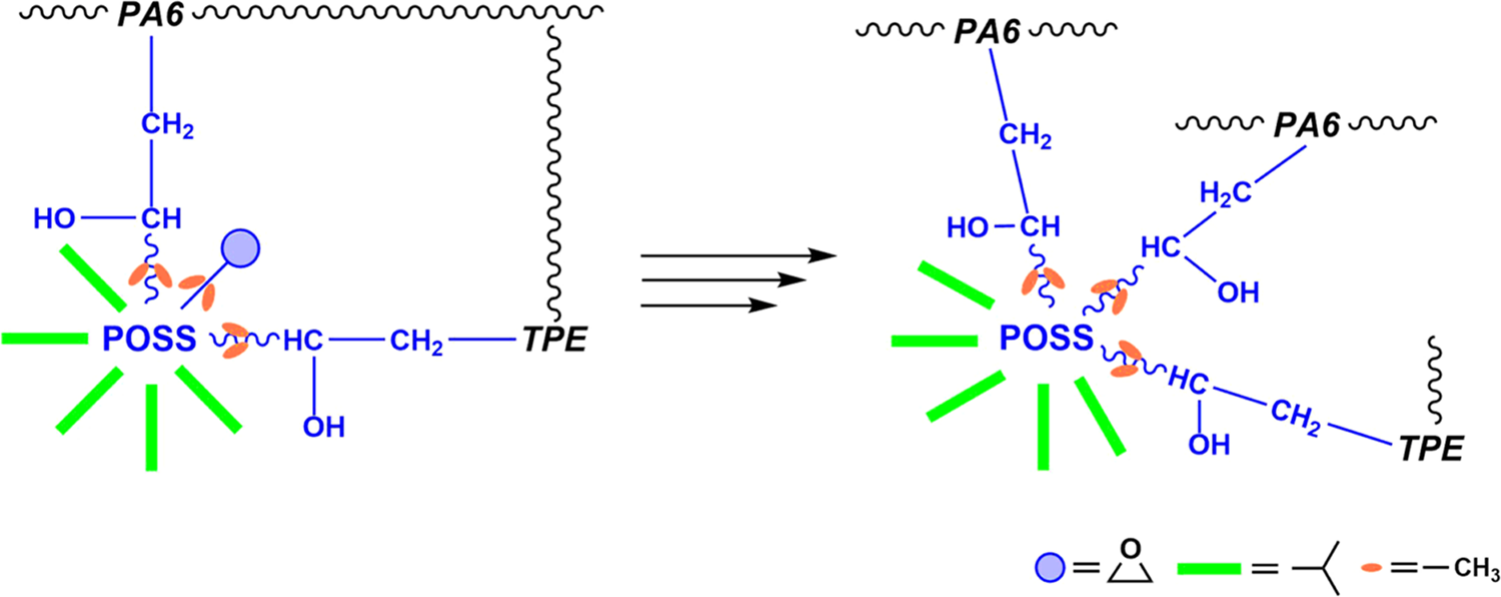
Formation of New Bonds as a Result of the Relaxation of the Side Groups of TriEpPOSS during the Repeated Extrusion Process

As obtained from rheological analyses, the overall rheological properties of PA6/TPE blends incorporating TriEpPOSS exhibited a rise with repeated extrusion cycles. This trend can be attributed to the steric hindrance imposed by the methyl and isobutyl groups within the structure of TriEpPOSS. Specifically, the third epoxy group, situated between two other epoxy groups in the TriEpPOSS structure, remained unreactive during the initial extrusion step; however, it may react with the functional groups of components in the PA6/TPE blend during subsequent extrusions (Scheme 2). During each extrusion cycle, chain scission reactions ensued, yet the unreacted epoxy groups facilitated the formation of high molecular weight chains upon bonding with PA6 and/or TPE. Consequently, the rheological properties, such as complex viscosity and storage modulus values increased with an increasing number of extrusions.^39^

Conversely, it was noted that the complex viscosity values of 80PA6/20TPE blends compatibilized with MultEpPOSS declined through repeated extrusion processes. This decline is attributed to the formation of low molecular weight short chains arising from the degradation of MultEpPOSS nanoparticles, which are susceptible to thermooxidative degradation via methylene groups. Additionally, the extended exposure to heat treatment during repeated extrusion processes induces degradation of both PA6 and TPE components, further contributing to the reduction in complex viscosity values (Scheme 2). On the other hand, MultEpPOSS compatibilized blends have still higher complex viscosity values than that of TriEpPOSS-compatibilized blends during each processing cycles since the molecular weight of PA6/TPE blend is much higher in the presence of MultEpPOSS after the first extrusion step due to higher reactivity of MultEpPOSS.

### 3.4 Tensile Properties

The repeated extrusion processes induce degradations that precipitate alterations in the internal architecture of the polymer, thereby manifesting discernible shifts in the material’s mechanical characteristics. To evaluate the effect of thermomechanical cycles on the mechanical properties of uncompatibilized and POSS compatibilized PA6/TPE blends, tensile tests were carried out.

**Figure 14:**
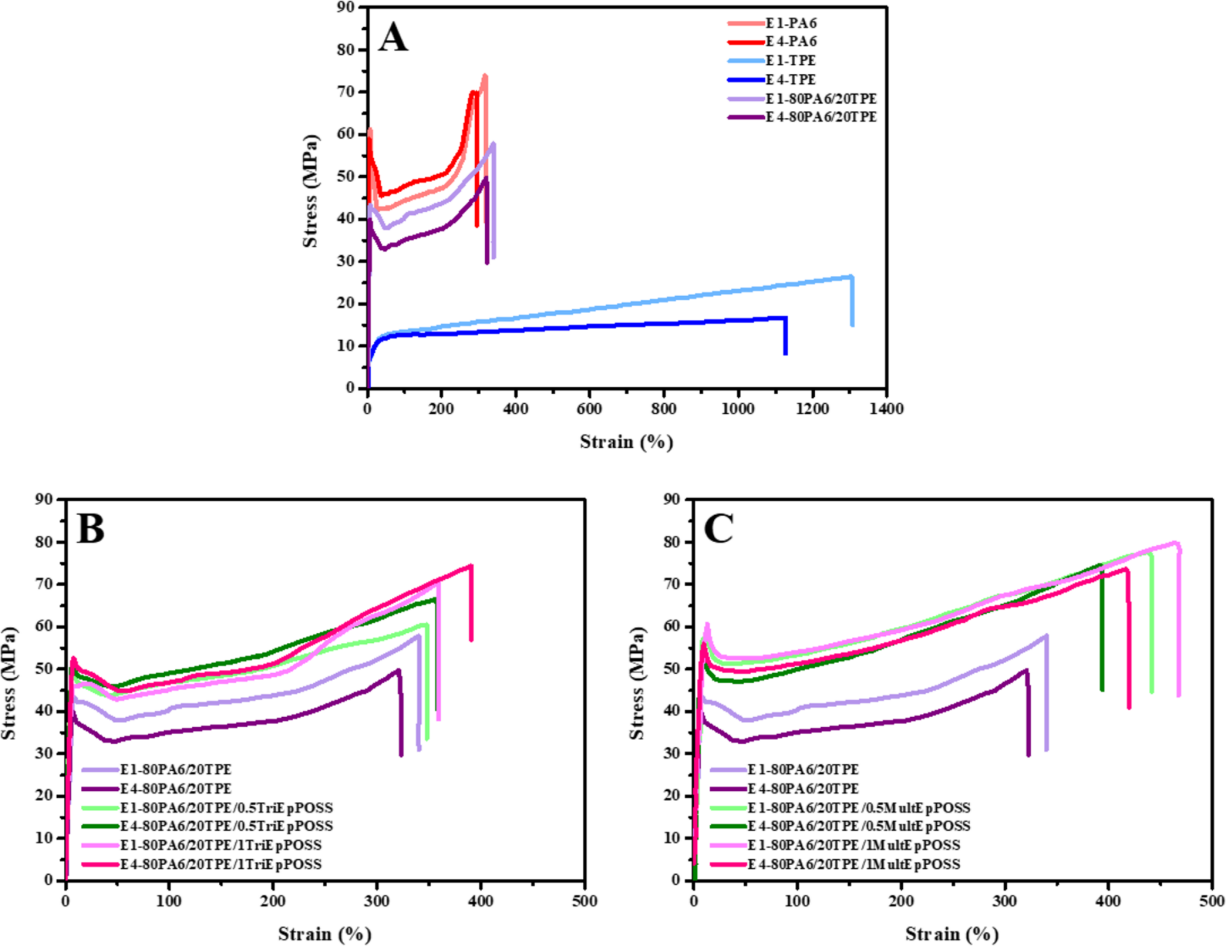
Stress–strain curves of samples after repeated extrusion process; (A) pure polymers and 80PA6/20TPE blend, (B) 80PA6/20TPE and 80PA6/20TPE/TriEpPOSS blends, (C) 80PA6/20TPE and 80PA6/20TPE/MultEpPOSS blends.

Figure 14 illustrates the stress–strain curves for repeatedly extruded PA6, TPE, and an 80PA6/20TPE blend, both compatibilized and uncompatibilized with POSSs. The E1-PA6 demonstrated necking behavior followed by cold drawing, with stress values increasing concomitantly with strain during the tensile test. This behavior can be attributed to the stress-induced crystallization of PA6. In contrast, TPE exhibited no necking behavior; instead, it displayed elastomeric characteristics due to the presence of flexible groups, resulting in failure at a higher elongation value.^40^ For the TPE-toughened PA6 blends, the observed stress–strain curves were similar to those of PA6. Notably, following repeated extrusion processes, the stress–strain behavior of E1-PA6, TPE, and the 80PA6/20TPE blend remained consistent; however, a decline in tensile properties was noted. As seen in Figure 14, the incorporation of TriEpPOSS and MultEpPOSS into the 80PA6/20TPE blend did not alter the stress–strain behavior, regardless of the number of extrusions. Conversely, the tensile properties of the blends exhibited significant enhancement, particularly with the addition of MultEpPOSS. Furthermore, the tensile strength of the PA6/TPE blend markedly increased with the addition of TriEpPOSS after repeated extrusion, as illustrated in Figure 14B.

**Figure 15:**
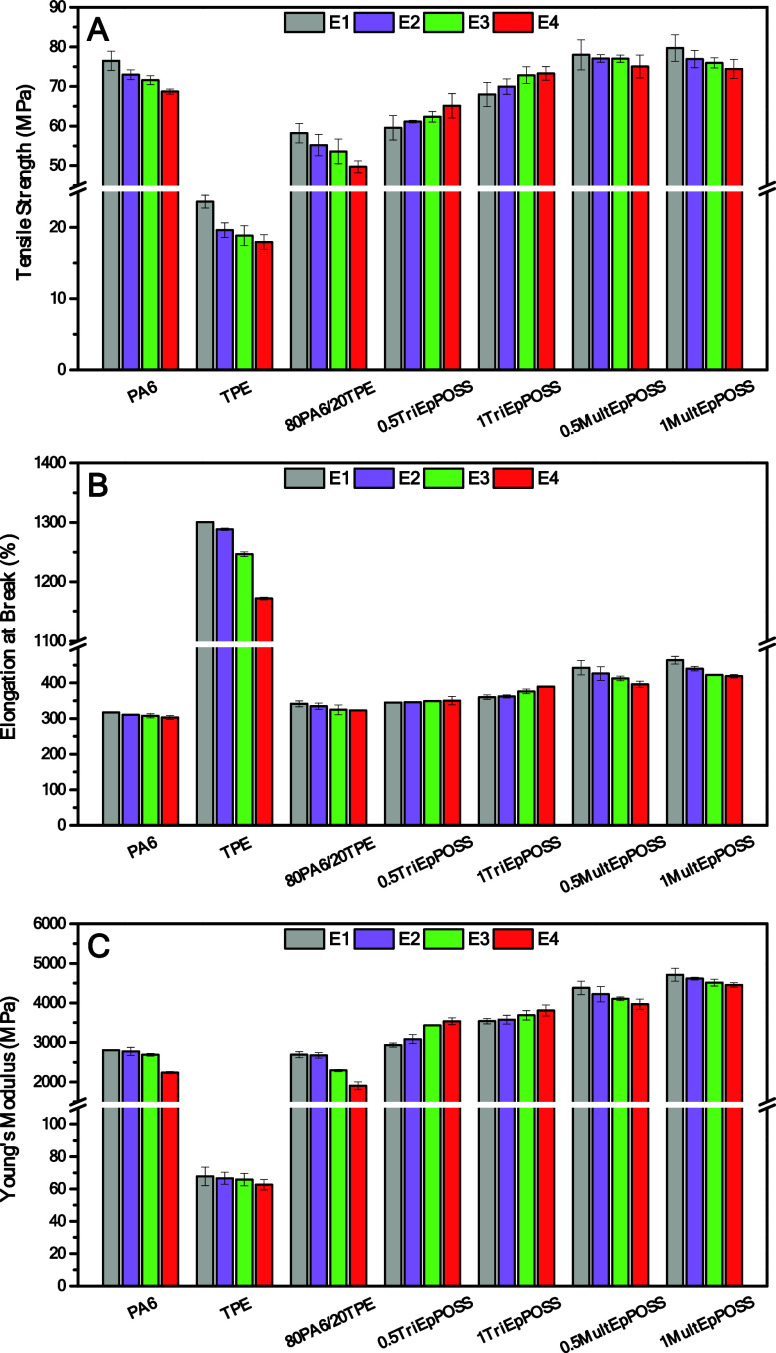
Changes in the tensile properties of samples after repeated extrusion process; (A) tensile strength, (B) elongation at break, (C) Young’s modulus.

As seen in Figure 15, all mechanical properties of pure PA6 and TPE systematically decreased as the extrusion cycle increased. This observation could be ascribed to the reduction in molecular weight, leading to the formation of polymer chains with shorter lengths. These shorter chains may exhibit enhanced packing within the free volume of the material, thereby mitigating the loss in mechanical properties stemming from thermomechanical degradation.^14^

The tensile strength of the E1-PA6 sample, measured at 76.5 MPa, showed a decrement to 58.2 MPa upon the introduction of 20 wt % TPE into pure PA6, as illustrated in Figure 15. Similarly, the Young’s modulus, initially recorded at 2806.2 MPa for E1-PA6, exhibited a reduction to 2694.7 MPa upon TPE incorporation. Same observations were also obtained for elongation at break values. This was due to the unstable phase morphology of PA6/TPE blends. Therefore, poor interfacial interaction between the components resulted in a decrease in the mechanical properties.^41−43^ It was observed that the mechanical properties of 80PA6/20TPE blends exhibited a declining trend with an increasing number of repeated extrusion cycles, as depicted in Figure 15. The decrease in tensile strength values upon repeated extrusion cycles can be attributed to the occurrence of chain scission reactions induced by prolonged exposure to thermal energy during the repeated extrusion processing, as elucidated in the rheological analysis findings. Furthermore, the reduction in elongation at break values correlates with an increase in crystallinity percentage due to the formation of shorter chains within the structure, coupled with a decrease in molecular weight resulting from chain scission reactions following prolonged thermal treatment.^44^ This enhanced chain mobility, thereby facilitating crystal formation. Therefore, crack propagation and early damage initiation beyond the elastic domain occurred. Similarly, the decline in Young’s modulus values with an escalating number of repeated extrusion cycles is presumed to be linked to an increase in the average particle size of the dispersed phase. The increased average particle size of the dispersed phase of TPE after repeated extrusion process created a lower interfacial area between the components; therefore, it adversely affected the stiffness of the material.

The tensile strength of E1-80PA6/20TPE blends exhibited increments from 58.2 to 68.0 and 79.7 MPa upon the incorporation of 1 wt % of TriEpPOSS and MultEpPOSS, respectively. Concurrently, enhancements were also observed in the elongation at break and Young’s modulus values following the addition of POSS, irrespective of the specific type employed. These advancements in mechanical properties stem from the interaction of POSS molecules through epoxy functional groups with the reactive groups present in the blend constituents. This interaction improved the interfacial adhesion and promoted the formation of a more stabilized phase morphology.^45−47^

Upon repeated extrusion cycles of 80PA6/20TPE blends incorporating TriEpPOSS, a proportional increment was observed in both tensile strength and Young’s modulus. For instance, the tensile strength of the E1-80PA6/20TPE/0.5TriEpPOSS blend increased from 59.6 to 65.1 MPa with an increase in extrusion cycles. Moreover, Young’s modulus enhanced from 2934.5 MPa in the E1-80PA6/20TPE/0.5TriEpPOSS sample to 3534.6 MPa after four repetitions of processing. These enhancements stem from the amelioration of the interface, facilitated by the reaction between the unreacted epoxy groups present in TriEpPOSS and the reactive groups inherent in PA6 and/or TPE, alongside a reduction in the average particle size of the dispersed phase during the further extrusion process. Furthermore, the elongation at break values exhibited an increase with the escalating number of extrusion cycles, attributed to the heightened chain entanglement of grafted and/or block copolymers formed within the interphase, consequently reducing the interfacial tension between PA6 and TPE.^48^ Conversely, in blends containing MultEpPOSS, a decrement in mechanical properties was observed with an increasing number of repeated extrusions, consistent with the rheological properties. As mentioned in SEM analyses, the average particle size of the dispersed phase increases upon repeated processing of 80PA6/20TPE/MultEpPOSS blends. Additionally, chain scission reactions induced by prolonged thermal treatment of the blends lead to a reduction in mechanical properties in the presence of MultEpPOSS.

### 3.5 Impact Test

To assess the impact strength of the samples, Izod impact strength tests were carried out on specimens with a 2 mm V-shaped notch.

**Figure 16:**
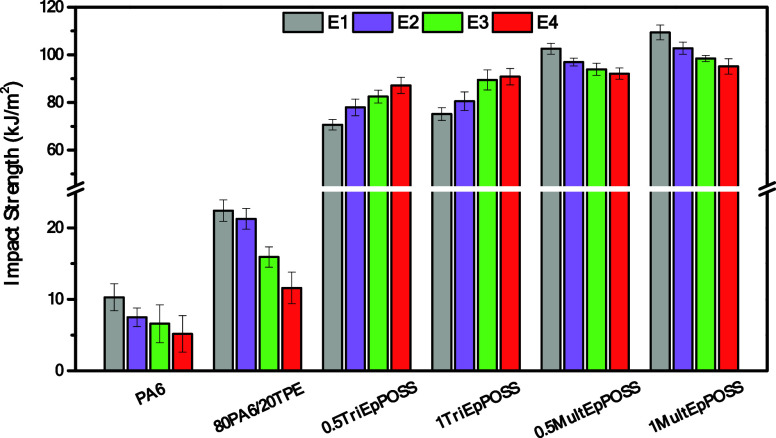
Changes in the Izod impact strength of PA6, TPE, 80PA6/20TPE and 80PA6/20TPE/POSS blends after repeated extrusion cycles.

Crystalline thermoplastics, such as PA6, are highly susceptible to cracking and crack propagation, resulting in relatively low toughness and notched impact resistance. Consequently, their limited impact and bending resistance, particularly at low temperatures and high deformation rates, restricts their suitability for many applications. Therefore, PA6 exhibited a low impact strength of ∼10 kJ/m^2^ due to its high crystallinity. To enhance the notch sensitivity of PA6, materials with high impact resistance, such as thermoplastic elastomers, are frequently utilized.^49,50^ As expected, the impact strength of the E1-PA6 sample exhibited a notable enhancement of 117% upon the incorporation of TPE, as illustrated in Figure 16. A systematic decline in Izod impact strength values was observed upon subjecting PA6 and 80PA6/20TPE to repeated extrusion processes. This was due to the fact that during polyamide recycling, the polymer chains undergo degradation due to elevated mechanical stresses and thermal influences. Consequently, this degradation instigates a reduction in the molecular weight of polyamides, thereby precipitating a corresponding deterioration in mechanical properties.^51^

Increases in Izod impact strength values of E1-80PA6/20TPE/POSS blends were determined compared to those without compatibilizer. For example, the Izod impact strength value of 22.4 kJ/m^2^ in the E1-80PA6-20TPE blend increased to 70.7and 75.2 kJ/m^2^ with the addition of 0.5 and 1% TriEpPOSS by weight, respectively. Moreover, as expected, the highest Izod impact strength values were obtained in the E1-80PA6/20TPE, including MultEpPOSS. This increment in Izod impact strength is more pronounced for 1 wt % MultEpPOSS incorporation. This was due to the higher reactivity of MultEpPOSS toward the components in PA/TPE blends since MultEpPOSS has eight functional epoxide groups on its cage structure. As widely acknowledged, the impact strength of polymer blends is intricately linked to the average particle size of the dispersed phase or, equivalently, its distribution within the matrix. The presence of homogeneous and smaller particles dispersed within the matrix engenders heightened toughness through the following reasons. First, stress fluctuations around the particle dimensions of the dispersed phase induce cavitations within the matrix, consequently leading to localized stress concentration and plastic deformation of the matrix.^52,53^ However, it is imperative that cavitated particles do not prompt the fracture process. Hence, the average particle size of these dispersed particles within the matrix must remain exceedingly small, thus mitigating the probability of crack initiation.^54^ As mentioned in SEM analyses, the dispersed particle size of TPE in 80PA6/20TPE blends was significantly reduced with the addition of POSSs nanoparticles, especially in the presence of MultEpPOSS. This results in enhanced interphase interaction between PA6 and TPE as a result of reactive compatibilization.^55,56^ Therefore, it can be concluded that the incorporation of POSSs nanoparticles served to elevate Izod impact strength values of the 80PA6/20TPE blend through a 2-fold mechanism during the first extrusion processing step: by increasing the molecular weight of the 80PA6/20TPE blend via the formation of the block and/or graft copolymers within the interphase of the components and by diminishing the average particle size of the dispersed phase of TPE. Consequently, this dual effect culminates a noteworthy enhancement in Izod impact strength values.^24^

For the Izod impact strength of PA6/TPE/TriEpPOSS blends after repeated extrusion cycles, a remarkable improvement in Izod impact strength values was acquired, as seen in Figure 16. The observed increments in Izod impact strength values within the 80PA6/20TPE samples, subjected to repetitive extrusion cycles, are conjectured to arise from the progressive enhancement of the interfacial properties in the presence of TriEpPOSS. This enhancement is postulated to occur during each extrusion step, facilitated by the reaction of unreacted epoxy groups in TriEpPOSS at initial extrusion steps, as delineated in Scheme 2. Therefore, it can be concluded that the interface between PA6 and TPE was re-established at each processing step in the presence of TriEpPOSS.^51,57,58^ Conversely, there is a drop in the toughness of 80PA6/TPE blends with the addition of MultEpPOSS as the extrusion cycles increase; however, it is still higher as a comparison with repeatedly extruded PA6/TPE/TriEpPOSS blends. This was due to the degradation of MultEpPOSS and the formation of carbonyl groups, as explained in detail in the FTIR analyses. Surprisingly, as the amount of MultEpPOSS nanoparticles increased in PA6/TPE blends, the Izod impact strength values exhibited higher values during repeated extrusion process. As detected in the SEM images, the 0.9 ± 0.1 μm average particle size of the dispersed phase of TPE in E4-80PA6/20TPE/0.5MultEpPOSS increased 1.5 ± 0.3 μm for the PA6/TPE blend, including 1 wt % MultEpPOSS. This showed that larger dispersed TPE particles increased the energy requirement for crack propagation by acting as a barrier.^59−61^ In this conceptual framework of crack pinning and bowing, initially presented by Lange^62^ and Evans,^63^ the interaction between a propagating crack and a secondary dispersed phase necessitates a greater energy expenditure compared to crack propagation within the unmodified material. The fracture energy requisite for advancing the crack in the presence of a dispersed second phase of TPE is amplified as the interparticle spacing diminishes. Consequently, a higher concentration of MultEpPOSS resulted in larger dispersed particles of TPE, which reduced distances between the TPE particles attributable to a more uniform dispersion, intensifying the demand for crack propagation.

### 3.6 Heat Deflection Temperature (HDT) Test

Table 3 shows the results of HDT tests conducted on pure PA6, TPE, uncompatibilized and POSS compatibilized 80PA6/20TPE blends subjected to the repeated extrusion process. As seen from Table 3, HDT values for E1-PA6 and E1-TPE samples under a load of 0.46 MPa were recorded at 57.5 and 61.2 °C, respectively. One of the criteria that affect the HDT of a polymeric material is its crystallinity values. The higher the percent crystallinity of a sample, the greater its tendency to show improved HDT. As shown in DSC results, pure TPE has higher crystallinity than PA6. Therefore, TPE exhibited a superior HDT value compared to PA6. The HDT assessments conducted on pure PA6, pure TPE, and their blends subjected to repeated extrusion cycles revealed a consistent trend of diminishing HDT values correlating with increasing extrusion cycles. This decline in HDT values can be attributed to the consequential reduction in the molecular weight of the polymers induced by prolonged exposure to heat treatment during extrusion processes. This molecular weight reduction stems from chain scission reactions, a manifestation of degradation processes, as corroborated by the rheological analysis findings.^64^

**Table 3:** HDT Values of Samples after the First and Fourth Reprocessing Cycles

**sample**	**HDT (°C)**
E1-PA6	57.5
E4-PA6	53.1
E1-TPE	61.2
E4-TPE	53.4
E1-80PA6/20TPE	92.5
E4-80PA6/20TPE	77.4
E1-80PA6/20TPE/1TriEpPOSS	57.5
E4-80PA6/20TPE/1TriEpPOSS	75.8
E1-80PA6/20TPE/1MultEpPOSS	59.8
E4-80PA6/20TPE/1MultEpPOSS	56.5

In addition, HDT values decreased with the addition of TriEpPOSS and MultEpPOSS to the E1-80PA6/20TPE blend. This is because the graft or block copolymers formed in the interphase with the addition of POSS to the structure prevent crystallization by restricting chain mobility.^65^ On the other hand, it was observed that the HDT values increased with repeated extrusion of the 80PA6/20TPE blend containing TriEpPOSS. This increase in HDT values is due to the fact that the repeated extrusion process leads to a decrease in the average particle size of the dispersed phase of TPE. With the reduction in the average particle size, the dispersed phase acts as a nucleating agent, providing new areas for the formation of crystals and causing an increase in the %-crystallinity value. Moreover, the HDT serves as an indicator of a material’s stiffness as temperature increases. Stiffness predominantly stems from the polymer chains’ capability to respond to applied loads. A polymeric material with higher chain entanglement, in other words, higher elasticity modulus, can display superior HDT values.^66^ As discussed in the mechanical test results, 80PA6/20TPE blend in the presence of TriEpPOSS revealed enhanced Young’s modulus after the fourth extrusion process, which was due to the increased molecular weight of the PA6/TPE blends, resulting from the formation of graft or block copolymers. The increase in %-crystallinity with the increase in the number of repeated extrusion processes leads to a much stiffer structure of the 80PA6/20TPE blend; therefore, it significantly enhanced HDT value with the addition of TriEpPOSS.^67^ Conversely, HDT values decreased with repeated extrusion of 80PA6/20TPE blends compatibilized with MultEpPOSS. This reduction in HDT value is due to the formation of low molecular weight chains as the number of extrusions increases and the decrease in Young’s modulus values, which is an indicator of stiffness.^68^

### 3.7 Differential Scanning Calorimeter (DSC)

**Figure 17:**
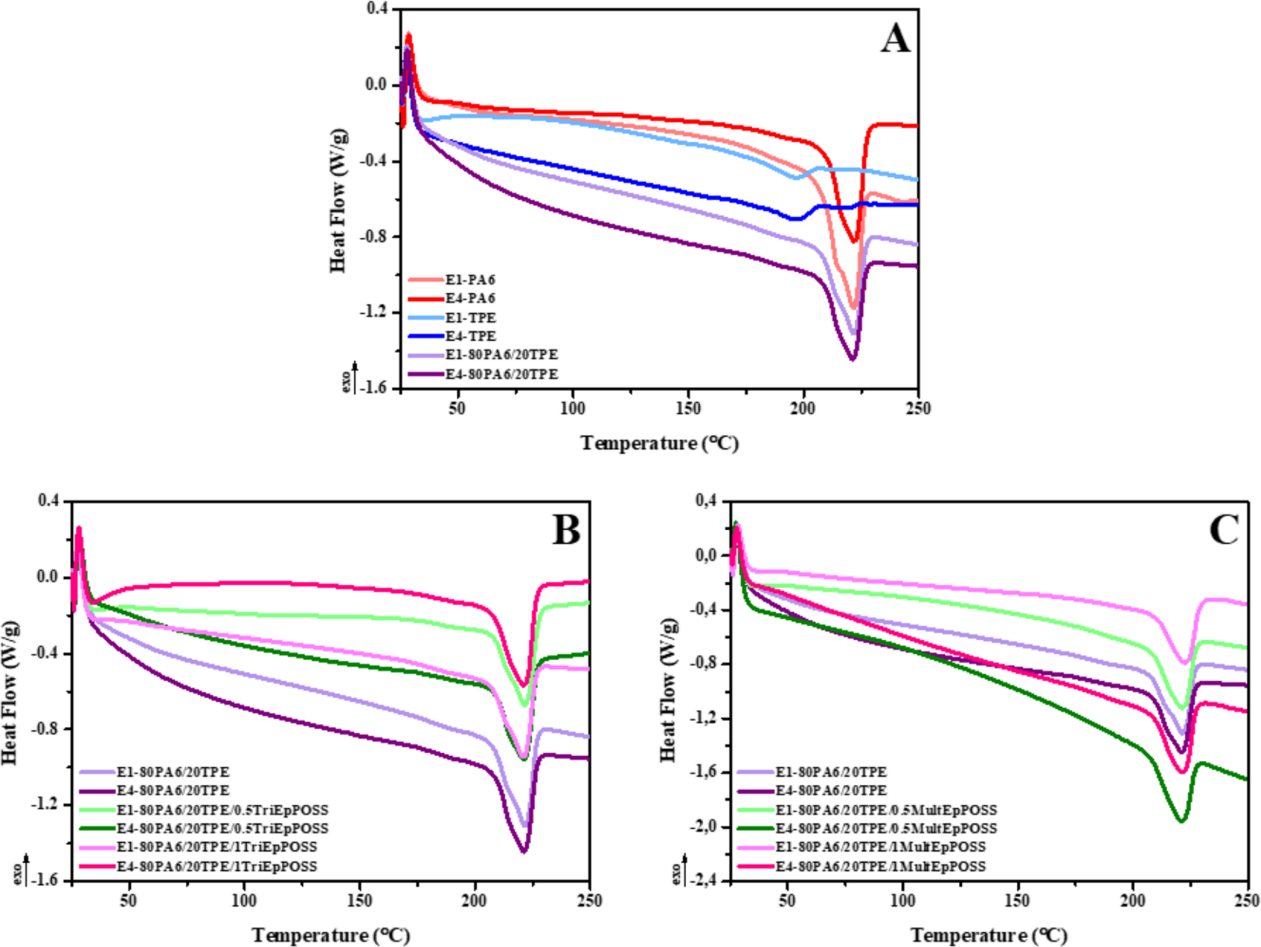
DSC thermograms of samples after the first and fourth reprocessing cycles; (A) pure polymers and 80PA6/20TPE blend, (B) 80PA6/20TPE and 80PA6/20TPE/TriEpPOSS blends, (C) 80PA6/20TPE and 80PA6/20TPE/MultEpPOSS blends.

The thermal properties of repeatedly extruded pure PA6 and TPE samples and 80PA6/20TPE blends with and without compatibilizer were investigated by DSC analyses. Figure 17 indicates the DSC thermograms of the samples. The thermal transitions obtained from DSC curves were tabulated in Table 4.

**Table 4:** Thermal Properties of Samples after the First and Fourth Reprocessing Cycles

**sample**	***T***_**m,TPE**_**(°C)**	**Δ***H*_**m,TPE**_ (J/g)	***X***_**c,TPE**_**(%)**
E1-TPE	196.0	13.1	41.6
E4-TPE	196.7	13.4	42.7

**sample**	***T***_**m,PA6**_**(°C)**	**Δ***H*_**m,PA6**_ (J/g)	***X***_**c,PA6**_**(%)**
E1-PA6	221.4	48.6	21.1
E4-PA6	221.8	60.2	26.2
E1-80PA6/20TPE	221.5	42.3	23.0
E4-80PA6/20TPE	221.3	47.6	25.8
E1-80PA6/20TPE/0.5TriEpPOSS	221.7	40.4	21.9
E4-80PA6/20TPE/0.5TriEpPOSS	221.1	53.7	29.2
E1-80PA6/20TPE/1TriEpPOSS	220.8	38.8	21.1
E4-80PA6/20TPE/1TriEpPOSS	221.2	50.7	27.5
E1-80PA6/20TPE/0.5MultEpPOSS	221.4	41.6	22.6
E4-80PA6/20TPE/0.5MultEpPOSS	220.8	47.5	25.8
E1-80PA6/20TPE/1MultEpPOSS	222.7	40.7	21.1
E4-80PA6/20TPE/1MultEpPOSS	221.4	48.6	26.4

The melting temperatures (*T*_m_) of E1-PA6 and E1-TPE were determined to be 221.4 and 196.0 °C, respectively. Additionally, the melting enthalpies (Δ*H*_m_) of E1-PA6 and E1-TPE were observed as 48.6 and 13.1 J/g, respectively (Table 4). This result is an indication that PA6, which has a higher melting enthalpy, contains spherulites with a more regular structure, which melts at higher temperatures, than TPE. The %-crystallinity (%*X*_c_) values of PA6, TPE, and PA6 in 80PA6/20TPE blends were determined by using 100% crystalline melting enthalpies of PA6 and TPE, reported as 230.0 J/g^69^ and 31.1 J/g,^70^ respectively. As seen in Table 4, TPE demonstrates a greater degree of crystallinity relative to PA6, thereby implying a higher density of spherulites.

The melting temperature of E1-PA6, which was found to be 221.4 °C, was observed to remain almost unchanged with the addition of 20 wt % of TPE to PA6. In addition, in the 80PA6/20TPE blend, the melting enthalpy of TPE was not observed since PA6 suppresses the crystallization of the dispersed phase TPE. Besides, the melting enthalpy of PA6 decreased from 48.6 to 42.3 J/g with the addition of 20 wt % TPE to E1-PA6. The observed phenomenon can be attributed to the transformation of spherulites within the structure of PA6 into a more irregular crystal morphology as the concentration of TPE increases. The calculated %*X*_c_ value of E1-PA6 slightly increased from 21.1 to 23.0% for the 80PA6/20TPE blend. This showed that the dispersed particles of TPE within the matrix facilitated the formation of new crystals of PA6, serving as a nucleating agent.^71^ It was observed that there was no significant change in the melting temperatures of repeatedly extruded PA6 and TPE samples and 80PA6/20TPE blends as a result of repeated extrusion. Nevertheless, the melting enthalpy and %*X*_c_ values exhibited an upward trend with an increasing number of repeated extrusions. This can be attributed to the formation of low molecular weight short polymer chains resulting from degradation reactions induced by the thermal energy exposed to the samples during reprocessing. The formation of these short chains enhances chain mobility and facilitates nucleation processes, thereby contributing to the observed increase in both melting enthalpy and %*X*_c_ values.^31,44,72−74^

The incorporation of POSSs into the E1-80PA6/20TPE blend, regardless of the POSS type, did not induce any significant alteration in the melting temperature of PA6. However, the addition of POSS to the E1-80PA6/20TPE blend, independent of POSS concentration, caused a decrease in melting enthalpy and %-crystallinity values (Table 4). This phenomenon stems from the interaction between the epoxy groups present in the structure of POSSs and the reactive functional groups of PA6 and TPE, as mentioned before. Consequently, the formation of long chains of the graft and/or block copolymers formed during reactive compatibilization diminished the folding of polymer chains, resulting in lower enthalpy of melting and %-crystallinity values.^75^

During the repeated extrusion of 80PA6/20TPE/TriEpPOSS blends, it was observed that melting temperatures remained nearly constant. However, an increase in melting enthalpy and %-crystallinity values was detected with an increasing number of repeated extrusions. As previously discussed, in the initial extrusion stage, the steric hindrance effect of the methyl and isobutyl groups in the cage structure of TriEpPOSS prevents the epoxy group between the two epoxy groups of TriEpPOSS from chemical interactions with PA6 and/or TPE. Accordingly, upon increasing the number of extrusion cycles, this unreacted epoxy group can react with components in PA6/TPE blend. Despite chain scission reactions occurring during repeated extrusion, the emergence of new graft and/or block copolymers within the interphase enhanced the molecular weight of the polymer blend. As a result, the average particle size of the dispersed phase of TPE significantly decreased; therefore, this smaller particles acted as heterogeneous nucleation agent zones, facilitating the crystallization of the continuous phase. Consequently, this leads to higher %-crystallinity and enthalpy of melting values. Similar to TriEpPOSS, the addition of MultEpPOSS did not induce significant changes in the melting temperature of PA6 in PA6/TPE blend. However, increasing the number of repeated processes resulted in heightened melting enthalpy and %-crystallinity values of PA6 in the presence of MultEpPOSS. This increment is attributed to the chain scission reactions that occurred in the blends during repeated extrusion processing. Consequently, the short chains generated as a result of these reactions facilitate crystal formation, act as nucleating agents, and contribute to the increase in melting enthalpy and %-crystallinity values.^76−78^

### 3.8 Thermogravimetric Analysis (TGA)

The thermal stability of pure PA6 and TPE samples, PA6/TPE blends with and without compatibilizer produced by repeated extrusion were investigated by TGA analysis. *T*_d5_, *T*_d10_, and *T*_dmax_, reported in Table 5, denote the temperatures at which 5%, 10%, and the maximum weight loss are observed during decomposition, respectively.

**Table 5:** TGA Analysis Results of Once and 4 Times Extruded Samples

**sample**	***T***_**d5**_**(°C)**	***T***_**d10**_**(°C)**	***T***_**dmax**_**(°C)**
E1-PA6	393.2	408.8	452.0
E4-PA6	387.1	406.5	452.4
E1-TPE	368.7	378.6	407.1
E4-TPE	371.4	379.8	405.8
E1-80PA6/20TPE	376.4	385.7	439.4
E4-80PA6/20TPE	370.6	380.5	435.2
E1-80PA6/20TPE/0.5TriEpPOSS	369.9	379.6	442.7
E4-80PA6/20TPE/0.5TriEpPOSS	370.3	380.5	439.8
E1-80PA6/20TPE/1TriEpPOSS	370.3	380.2	442.4
E4-80PA6/20TPE/1TriEpPOSS	373.4	384.4	449.4
E1-80PA6/20TPE/0.5MultEpPOSS	373.2	381.7	441.1
E4-80PA6/20TPE/0.5MultEpPOSS	374.4	383.5	440.6
E1-80PA6/20TPE/1MultEpPOSS	369.4	382.4	439.4
E4-80PA6/20TPE/1MultEpPOSS	363.3	377.9	439.4

**Figure 18:**
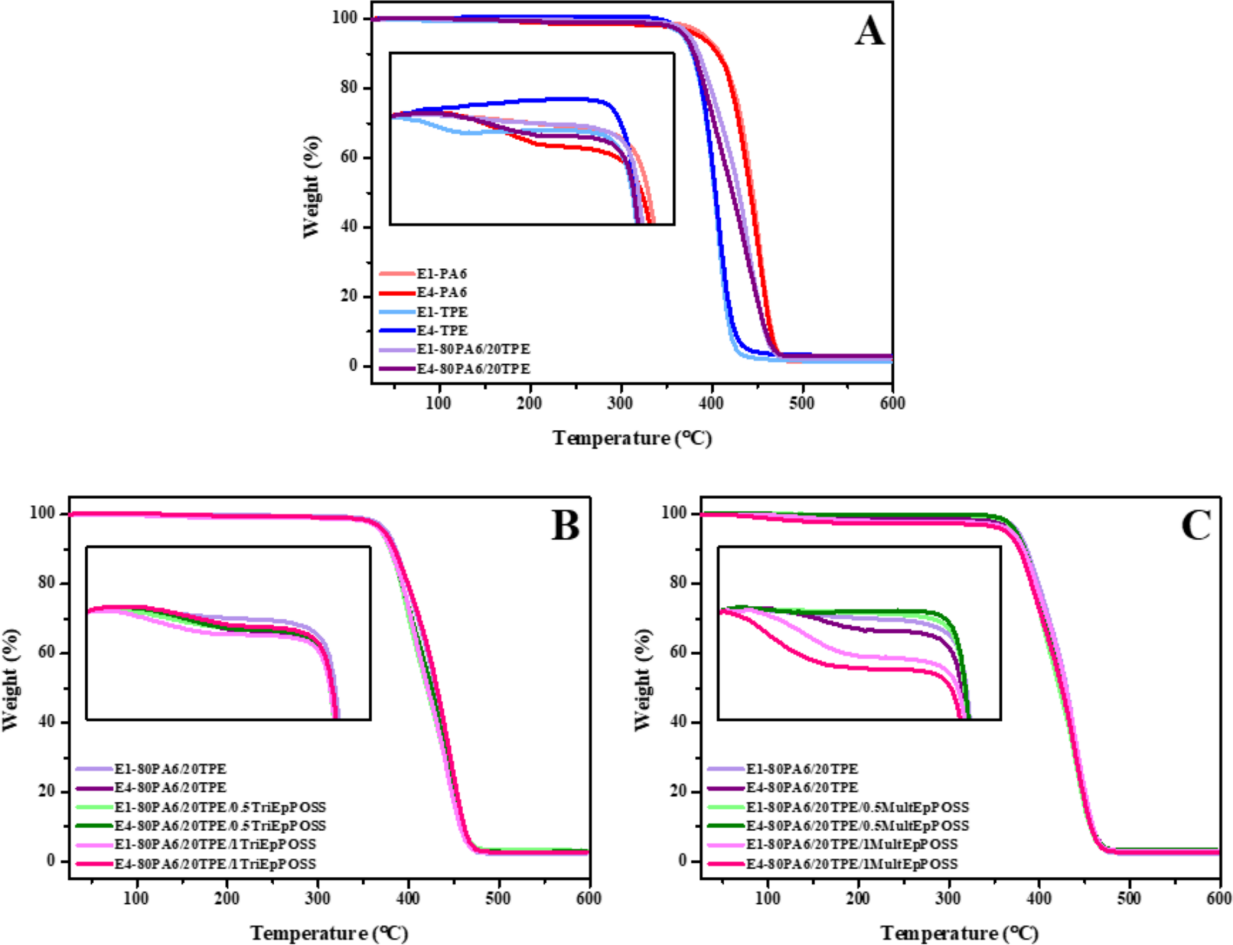
TGA curves of samples after the first and fourth reprocessing cycles; (A) pure PA6, TPE and 80PA6/20TPE blend, (B) 80PA6/20TPE and 80PA6/20TPE/TriEpPOSS blends, (C) 80PA6/20TPE and 80PA6/20TPE/MultEpPOSS blends.

The thermal degradation of E1-PA6 started at approximately 393.0 °C and ended at 468.0 °C, whereas the degradation of E1-TPE initiated at 386.4 °C and terminated at 419.9 °C. Notably, both samples demonstrated a characteristic single-step degradation behavior, as depicted in Figure 18A. These findings conclusively establish the higher thermal stability of E1-PA6 relative to E1-TPE. Concerning the thermal stability of PA6 samples, it was observed that the degradation temperatures of *T*_d5 and_*T*_d10_ decreased with an increase in the number of repeated extrusions. These reductions can be attributed to the generation of lower molecular weight chains as a consequence of chain scission reactions occurring during reprocessing. The decline in chain molecular weight entails a diminished energy threshold necessary for thermal decomposition.^1,31,79,80^ The degradation behavior of pure TPE samples following repeated extrusion exhibited dissimilarities compared to pure PA6. Specifically, it was observed that the *T*_d5 and_*T*_d10_ values remained nearly constant despite prolonged exposure to thermal energy. This observation suggests that the thermal stability of the polyester-based TPE after repeated extrusion is higher than that of pure PA6. The TGA analysis of 80PA6/20TPE blends revealed a trend where the *T*_d5 and_*T*_d10_ values progressively decreased with an increasing number of repeated extrusions. This observation indicates that PA6 may contribute to a decrease in the thermal stability of TPE as the number of reprocessing cycles increases.

The decline in thermal stability observed in the 80PA6/20TPE blend with an increasing number of repeated extrusions began to exhibit a reverse trend upon the addition of TriEpPOSS nanoparticles to the blend. For instance, the *T*_d5_ value of the E1-80PA6/20TPE blend, initially at 376.4 °C, decreased to 370.6 °C for the E4-80PA6/20TPE sample, reflecting a reduction in molecular weight. However, in contrast, the *T*_d5_ value of the E1-80PA6/20TPE/1TriEpPOSS blend, initially at 370.3 °C, increased to 373.4 °C for the E4-80PA6/20TPE/1TriEpPOSS blend. Likewise, *T*_dmax_ exhibited a notable increase of 7 °C, rising from 442.4 to 449.4 °C upon the addition of TriEpPOSS to the 80PA6/20TPE blend. The introduction of TriEpPOSS during the repeated extrusion phase led to a reduction in the size of TPE particles within the PA6/TPE blend. This reduction facilitates their homogeneous distribution throughout the matrix. These TPE particles with higher thermal stability and the silica present in the cage structure of POSS acted as a physical barrier against heat flux, consequently enhancing the thermal stability of the samples. Conversely, the thermal stability of the 80PA6/20TPE blend in the presence of MultEpPOSS decreased importantly during the repeated extrusion process. As previously mentioned, MultEpPOSS is considerably more susceptible to thermooxidative degradation, which catalyzed the degradation reactions of both PA6 and TPE.^28^

## 4 Conclusions

This study examines the reprocessing capability of PA6, TPE, 80PA6/20TPE blend, and PA6/TPE blends compatibilized with POSS molecules having different numbers of epoxide functional groups and loading ratios. The investigation involves exploring the alterations in thermal, morphological, rheological, and mechanical properties and potential interactions among the constituents.

The FTIR analysis results indicated the formation of carbonyl groups with chromophore properties as the number of extrusions increased, leading to a noticeable change in sample color. Moreover, FTIR analyses revealed the partial degradation of MultEpPOSS during reprocessing. Conversely, PA6/TPE blends, incorporating MultEpPOSS, demonstrated superior mechanical properties compared to uncompatibilized blends following the repeated extrusion process. SEM analyses demonstrated a decrease in the average dispersed particle size of TPE with an increasing reprocessing cycle in PA6/TPE blends compatibilized with TriEpPOSS. Regarding rheological properties, complex viscosity, storage modulus, and loss modulus of all samples decreased due to chain scission reactions with repeated extrusions, except for TriEpPOSS-compatibilized blends. Repeated extrusion led to an increase in both the enthalpy of melting and the %-crystallinity values across all samples. Adding TriEpPOSS nanoparticles with three epoxide functional groups on their cage structure significantly enhanced the mechanical and thermomechanical properties and the thermal stability of PA6/TPE blends.

In general, the incorporation of TriEpPOSS nanoparticles restored the morphology of the PA6/TPE blend, stabilized its structure against potential damage during forming, enhanced adhesion between PA6 and TPE in the solid state, and recompatibilized thermodynamically immiscible PA6/TPE blend acted as emulsifier during repeated processing. This finding holds significant importance in stabilizing PA6/TPE blends for recycling purposes. It presents a promising approach to mitigate the degradability of PA6/TPE blends and consequently enhance their recyclability. Considering the environmental sensitivities in the past decade, these POSS-containing materials have potential applications in the automotive industry in underhood applications covering the engine compartment of the vehicle, such as intake manifolds, flaps, and engine compartment covers.

## Supporting Information Available

FTIR spectra of PA6, TPE, 80PA6/20TPE and 80PA6/20TPE blends containing POSS nanoparticles after repeated extrusion cycles (PDF)

## References

[ref1] Domingo G. D.; Souza A. M. C. PA6/PA66/Talc Composite: Effect of Reprocessing on the Structure and Properties. J. Appl. Polym. Sci. 2022, 139 (13), 51869 10.1002/app.51869.

[ref2] Kudva R. A.; Keskkula H.; Paul D. R. Properties of Compatibilized Nylon 6/ABS Blends: Part I. Effect of ABS Type. Polymer 2000, 41 (1), 225–237. 10.1016/S0032-3861(99)00105-6.

[ref3] Harper C. A.; Petrie E. M. Plastics Materials and Processes, 1st ed.; John Wiley & Sons, Inc.: USA, 2003; Vol. 20 10.1109/mei.2004.1318854.

[ref4] Ajitha A. R.; Thomas S. Compatibilization of Polymer Blends:Micro and Nano Scale Phase Morphologies, Interphase Characterization and Properties; Ajitha A. R.; Thomas S., Eds.; Elsevier, 2020. 10.1016/B978-0-12-816006-0.00019-0.

[ref5] Ajji A.; Utracki L. A. Interphase and Compatibilization of Polymer Blends. Polym. Eng. Sci. 1996, 36 (12), 1574–1585. 10.1002/pen.10554.

[ref6] Stuart B. H. Polymer Analysis; Stuart B. H., Ed.; John Wiley & Sons, Inc: England, 2002. 10.1002/9780470511343.

[ref7] Thomas S.; Somasekharan L. Polyhedral Oligomeric Silsesquioxane (POSS) Polymer Nanocomposites: From Synthesis to Applications; Thomas S.; Somasekharan L., Eds.; Elsevier, 2021. 10.3390/NANO2040445.

[ref8] Zhou H.; Ye Q.; Xu J. Polyhedral Oligomeric Silsesquioxane-Based Hybrid Materials and Their Applications. Mater. Chem. Front. 2017, 1 (2), 212–230. 10.1039/C6QM00062B.

[ref9] Mehat N. M.; Kamaruddin S. Optimization of Mechanical Properties of Recycled Plastic Products via Optimal Processing Parameters Using the Taguchi Method. J. Mater. Process. Technol. 2011, 211 (12), 1989–1994. 10.1016/j.jmatprotec.2011.06.014.

[ref10] Kuram E.; Sarac L.; Ozcelik B.; Yilmaz F. Mechanical, Chemical, Thermal, and Rheological Properties of Recycled PA6/ABS Binary and PA6/PA66/ABS Ternary Blends. J. Appl. Polym. Sci. 2014, 131 (18), 40810 10.1002/app.40810.

[ref11] Francis R. Recycling of Polymers; Francis R., Ed.; Wiley, 2016; Vol. 10 10.1016/0361-3658(87)90003-8.

[ref12] Kudva R. A.; Keskkula H.; Paul D. R. Properties of Compatibilized Nylon 6/ABS Blends: Part II. Effects of Compatibilizer Type and Processing History. Polymer 2000, 41 (1), 239–258. 10.1016/S0032-3861(99)00106-8.

[ref13] La Mantia F. P.; Capizzi L. Recycling of Compatibilized and Uncompatibilized Nylon/Polypropylene Blends. Polym. Degrad. Stab. 2001, 71 (2), 285–291. 10.1016/S0141-3910(00)00177-4.

[ref14] Chikh A.; Benhamida A.; Kaci M.; Bourmaud A.; Bruzaud S. Recyclability Assessment of Poly(3-Hydroxybutyrate-Co-3-Hydroxyvalerate)/Poly(Butylene Succinate) Blends: Combined Influence of Sepiolite and Compatibilizer. Polym. Degrad. Stab. 2017, 142, 234–243. 10.1016/j.polymdegradstab.2017.07.014.

[ref15] Gröning M.; Hakkarainen M. Headspace Solid-Phase Microextraction with Gas Chromatography/Mass Spectrometry Reveals a Correlation between the Degradation Product Pattern and Changes in the Mechanical Properties during the Thermooxidation of in-Plant Recycled Polyamide 6,6. J. Appl. Polym. Sci. 2002, 86 (13), 3396–3407. 10.1002/app.11345.

[ref16] Singh B.; Sharma N. Mechanistic Implications of Plastic Degradation. Polym. Degrad. Stab. 2008, 93 (3), 561–584. 10.1016/j.polymdegradstab.2007.11.008.

[ref17] Sirin H.; Tuna B.; Ozkoc G. The Effects of Thermomechanical Cycles on the Properties of PLA/TPS Blends. Adv. Polym. Technol. 2014, 33 (S1), 21458 10.1002/adv.21458.

[ref18] Awaja F.; Pavel D. Recycling of PET. Eur. Polym. J. 2005, 41 (7), 1453–1477. 10.1016/j.eurpolymj.2005.02.005.

[ref19] Romão W.; Franco M. F.; Corilo Y. E.; Eberlin M. N.; Spinacé M. A. S.; De Paoli M. A. Poly (Ethylene Terephthalate) Thermo-Mechanical and Thermo-Oxidative Degradation Mechanisms. Polym. Degrad. Stab. 2009, 94 (10), 1849–1859. 10.1016/j.polymdegradstab.2009.05.017.

[ref20] Grigg M. N. Thermo-Oxidative Degradation of Polyamide 6; Queensland University of Technology, 2006.

[ref21] Marechal P.; Legras R.; Dekoninck J. M. Postcondensation and Oxidation Processes in Molten Polyamide 6. J. Polym. Sci., Part A: Polym. Chem. 1993, 31 (8), 2057–2067. 10.1002/pola.1993.080310812.

[ref22] Santos A. S. F.; Agnelli J. A. M.; Trevisan D. W.; Manrich S. Degradation and Stabilization of Polyolefins from Municipal Plastic Waste during Multiple Extrusions under Different Reprocessing Conditions. Polym. Degrad. Stab. 2002, 77 (3), 441–447. 10.1016/S0141-3910(02)00101-5.

[ref23] Xiang Q.; Xanthos M.; Mitra S.; Patel S. H.; Guo J. Effects of Melt Reprocessing on Volatile Emissions and Structural/Rheological Changes of Unstabilized Polypropylene. Polym. Degrad. Stab. 2002, 77 (1), 93–102. 10.1016/S0141-3910(02)00083-6.

[ref24] Yıldırım R.; Ullah M. S.; Koçoğlu H.; Ün M.; Yazıcı Çakır N.; Demir G.; Çetin D.; Urtekin G.; Özkoç G.; Mert O.; Kodal M. Effects of Hybrid POSS Nanoparticles on the Properties of Thermoplastic Elastomer-Toughened Polyamide 6. ACS Omega 2023, 8 (49), 47034–47050. 10.1021/acsomega.3c06896. 38107884 PMC10720298

[ref25] Chen R. S.; Ghani M. H. A.; Salleh M. N.; Ahmad S.; Gan S.; Chen R. S.; Ghani M. H. A.; Salleh M. N.; Ahmad S.; Gan S. Influence of Blend Composition and Compatibilizer on Mechanical and Morphological Properties of Recycled HDPE/PET Blends. Mater. Sci. Appl. 2014, 05 (13), 943–952. 10.4236/MSA.2014.513096.

[ref26] De Souza A. M. C.; Caldeira C. B. An Investigation on Recycled PET/PP and Recycled PET/PP-EP Compatibilized Blends: Rheological, Morphological, and Mechanical Properties. J. Appl. Polym. Sci. 2015, 132 (17), 41892 10.1002/app.41892.

[ref27] Pliquet M.; Rapeaux M.; Delange F.; Bussiere P. O.; Therias S.; Gardette J. L. Multiscale Analysis of the Thermal Degradation of Polyamide 6,6: Correlating Chemical Structure to Mechanical Properties. Polym. Degrad. Stab. 2021, 185, 109496 10.1016/j.polymdegradstab.2021.109496.

[ref28] Vilà Ramirez N.; Sanchez-Soto M.; Illescas S. Enhancement of POM Thermooxidation Resistance through POSS Nanoparticles. Polym. Compos. 2011, 32 (10), 1584–1592. 10.1002/pc.21191.

[ref29] Utracki L. A. Polymer Blends Handbook; Kluwer Academic Publishers: The Netherlands, 2002.

[ref30] Kiziltas A.; Nazari B.; Gardner D. J.; Bousfield D. W. Polyamide 6–Cellulose Composites: Effect of Cellulose Composition on Melt Rheology and Crystallization Behavior. Polym. Eng. Sci. 2014, 54 (4), 739–746. 10.1002/pen.23603.

[ref31] Crespo J. E.; Parres F.; Peydrõ M. A.; Navarro R. Study of Rheological, Thermal, and Mechanical Behavior of Reprocessed Polyamide 6. Polym. Eng. Sci. 2013, 53 (4), 679–688. 10.1002/pen.23307.

[ref32] Jiang R.; Chen Y.; Yao S.; Liu T.; Xu Z.; Park C. B.; Zhao L. Preparation and Characterization of High Melt Strength Thermoplastic Polyester Elastomer with Different Topological Structure Using a Two-Step Functional Group Reaction. Polymer 2019, 179, 121628 10.1016/j.polymer.2019.121628.

[ref33] López M. d. M. C.; Ares Pernas A. I.; Abad López M. J.; Latorre A. L.; López Vilariño J. M.; González Rodríguez M. V. Assessing Changes on Poly(Ethylene Terephthalate) Properties after Recycling: Mechanical Recycling in Laboratory versus Postconsumer Recycled Material. Mater. Chem. Phys. 2014, 147 (3), 884–894. 10.1016/j.matchemphys.2014.06.034.

[ref34] Farias N. C.; Major I.; Devine D.; Brennan Fournet M.; Pezzoli R.; Farshbaf Taghinezhad S.; Hesabi M. Multiple Recycling of a PLA/PHB Biopolymer Blend for Sustainable Packaging Applications: Rheology-Morphology, Thermal, and Mechanical Performance Analysis. Polym. Eng. Sci. 2022, 62 (6), 1764–1774. 10.1002/pen.25962.

[ref35] Dogan S. K.; Reyes E. A.; Rastogi S.; Ozkoc G. Reactive Compatibilization of PLA/TPU Blends with a Diisocyanate. J. Appl. Polym. Sci. 2014, 131 (10), 40251 10.1002/app.40251.

[ref36] Butnaru I.; Fernández-Ronco M. P.; Czech-Polak J.; Heneczkowski M.; Bruma M.; Gaan S. Effect of Meltable Triazine-DOPO Additive on Rheological, Mechanical, and Flammability Properties of PA6. Polymers 2015, 7 (8), 1541–1563. 10.3390/polym7081469.

[ref37] Kilic N. T.; Can B. N.; Kodal M.; Ozkoc G. The Potential Use of Epoxy-POSS as a Reactive Hybrid Compatibilizers for PLA/PBAT Blends: “Effect of PBAT Molecular Weight and POSS Type.. Polym. Eng. Sci. 2020, 60 (2), 398–413. 10.1002/pen.25295.

[ref38] Saeed Ullah M.; Yildirim R.; Caraseva L.; Zuza E.; Ozkoc G.; Kodal M. Miscibility and Phase Behavior of Reactively Compatibilized Poly(Lactic Acid)/Poly(Butylene Succinate) Bio-Blends Using Various Rheological Analyses. J. Appl. Polym. Sci. 2023, 140 (38), e54424 10.1002/app.54424.

[ref39] Wang R.; Wang S.; Zhang Y. Morphology, Rheological Behavior, and Thermal Stability of PLA/PBSA/POSS Composites. J. Appl. Polym. Sci. 2009, 113 (5), 3095–3102. 10.1002/app.30333.

[ref40] Wang L.; Guo L.; Zhang K.; Xia Y.; Hao J.; Wang X. Development of Tough Thermoplastic Elastomers by Leveraging Rigid–Flexible Supramolecular Segment Interplays. Angew. Chem., Int. Ed. 2023, 62 (29), e202301762 10.1002/anie.202301762. 37208825

[ref41] Kim Y. G.; Kim Y.; Choi J. K.; Baeck S. H.; Shim S. E. Preparation and Properties of Polypropylene/Thermoplastic Polyester Elastomer Blends. Polymer (Korea) 2017, 41 (3), 514–523. 10.7317/pk.2017.41.3.514.

[ref42] Naderi G.; Razavi-Nouri M.; Taghizadeh E.; Lafleur P. G.; Dubois C. Preparation of Thermoplastic Elastomer Nanocomposites Based on Polyamide-6/Polyepichlorohydrin-Co-Ethylene Oxide. Polym. Eng. Sci. 2011, 51 (2), 278–284. 10.1002/pen.21824.

[ref43] Mousavi M. R.; Tehran A. C.; Shelesh-Nezhad K. Study on Morphology, Mechanical, Thermal and Viscoelastic Properties of PA6/TPU/CNT Nanocomposites. Plast., Rubber Compos. 2020, 49 (9), 400–413. 10.1080/14658011.2020.1784588.

[ref44] Ayadi A.; Kraiem D.; Bradai C.; Pimbert S. Recycling Effect on Mechanical Behavior of HDPE/Glass Fibers at Low Concentrations. J. Thermoplast. Compos. Mater. 2012, 25 (5), 523–536. 10.1177/0892705711411343.

[ref45] Ismail H. H.; Nasir M. The Effect of Various Compatibilizers on Mechanical Properties of Polystyrene/Polypropylene Blend. Polym. Test. 2002, 21 (2), 163–170. 10.1016/S0142-9418(01)00064-2.

[ref46] Lee S. Y.; Kim S. C. Effect of Compatibilizer on the Crystallization, Rheological, and Tensile Properties of LDPE /EVOH Blends. J. Appl. Polym. Sci. 1998, 68, 1245–1256. 10.1002/(SICI)1097-4628(19980523)68:8.

[ref47] Ullah M. S.; Yildirim R.; Kodal M.; Ozkoc G. Reactive Compatibilization of PLA/PBS Bio-Blends via a New Generation of Hybrid Nanoparticles. J. Vinyl Addit. Technol. 2023, 29 (4), 737–757. 10.1002/vnl.21969.

[ref48] Karaagac E.; Koch T.; Archodoulaki V. M. The Effect of PP Contamination in Recycled High-Density Polyethylene (RPE-HD) from Post-Consumer Bottle Waste and Their Compatibilization with Olefin Block Copolymer (OBC). Waste Manage. 2021, 119, 285–294. 10.1016/j.wasman.2020.10.011. 33125937

[ref49] Dermanaki Farahani R.; Ramazani S A A. Melt Preparation and Investigation of Properties of Toughened Polyamide 66 with SEBS-g-MA and Their Nanocomposites. Mater. Des. 2008, 29 (1), 105–111. 10.1016/J.MATDES.2006.11.018.

[ref50] Muratoglu O. K.; Argon A. S.; Cohen R. E.; Weinberg M. Toughening Mechanism of Rubber-Modified Polyamides. Polymer 1995, 36 (5), 921–930. 10.1016/0032-3861(95)93590-I.

[ref51] Ozmen S. C.; Ozkoc G.; Serhatli I. E. Effect of Reactive Extrusion Process Parameters on Thermal, Mechanical, and Physical Properties of Recycled Polyamide-6: Comparison of Two Novel Chain Extenders. J. Macromol. Sci., Part B 2021, 60 (5), 350–367. 10.1080/00222348.2020.1852706.

[ref52] Bucknall C. B. Toughened Plastics; Bucknall C. B., Ed.; Applied Science Publishers, 1977. 10.1007/978-94-017-5349-4.

[ref53] Huang J. J.; Paul D. R. Comparison of Fracture Behavior of Nylon 6 versus an Amorphous Polyamide Toughened with Maleated Poly(Ethylene-1-Octene) Elastomers. Polymer 2006, 47 (10), 3505–3519. 10.1016/j.polymer.2006.03.038.

[ref54] Tang T.; Huang B. Interfacial Behaviour of Compatibilizers in Polymer Blends. Polymer 1994, 35 (2), 281–285. 10.1016/0032-3861(94)90691-2.

[ref55] Agrawal P.; Rodrigues A. W. B.; Araújo E. M.; Mélo T. J. A. Influence of Reactive Compatibilizers on the Rheometrical and Mechanical Properties of PA6/LDPE and PA6/HDPE Blends. J. Mater. Sci. 2010, 45 (2), 496–502. 10.1007/s10853-009-3967-9.

[ref56] Yilmaz S.; Gul O.; Yilmaz T. Effect of Chain Extender and Terpolymers on Tensile and Fracture Properties of Polyamide 6. Polymer 2015, 65, 63–71. 10.1016/j.polymer.2015.03.057.

[ref57] Murphy J. Additives for Plastics Handbooks; Elsevier Science, 2001.

[ref58] Gleria M.; Po R.; Giannotta G.; Fiocca L.; Bertani R.; Fambri L.; La Mantia F. P.; Scaffaro R. Cyclophosphazenes as Polymer Modifiers. Macromol. Symp. 2003, 196 (1), 249–270. 10.1002/masy.200390165.

[ref59] Ash B. J.; Siegel R. W.; Schadler L. S. Mechanical Behavior of Alumina/Poly(Methyl Methacrylate) Nanocomposites. Macromolecules 2004, 37 (4), 1358–1369. 10.1021/ma0354400.

[ref60] Milliman H. W.; Sánchez-Soto M.; Arostegui A.; Schiraldi D. A. Structure–Property Evaluation of Trisilanolphenyl POSS/Polysulfone Composites as a Guide to POSS Melt Blending. J. Appl. Polym. Sci. 2012, 125 (4), 2914–2919. 10.1002/app.36229.

[ref61] Kodal M.; Sirin H.; Ozkoc G. Effects of Reactive and Nonreactive POSS Types on the Mechanical, Thermal, and Morphological Properties of Plasticized Poly(Lactic Acid). Polym. Eng. Sci. 2014, 54 (2), 264–275. 10.1002/pen.23557.

[ref62] Lange F. F. The Interaction of a Crack Front with a Second-Phase Dispersion. Philos. Mag. 1970, 22 (179), 0983–0992. 10.1080/14786437008221068.

[ref63] Evans A. G. The Strength of Brittle Materials Containing Second Phase Dispersions. Philos. Mag. 1972, 26 (6), 1327–1344. 10.1080/14786437208220346.

[ref64] Aussawasathien D.; Prakymoramas N.; Thanomjitr D. Effects of Reprocessing on the Structure and Properties of Polycarbonate/Multi-Walled Carbon Nanotube Based Electrostatic Dissipative Composites. Chiang Mai J. Sci. 2013, 40 (2), 261–273.

[ref65] Reddy J. P.; Misra M.; Mohanty A. Injection Moulded Biocomposites from Oat Hull and Polypropylene/Polylactide Blend: Fabrication and Performance Evaluation. Adv. Mech. Eng. 2013, 5, 761840 10.1155/2013/761840.

[ref66] Ozmen S. C.; Ozkoc G.; Serhatli E. Thermal, Mechanical and Physical Properties of Chain Extended Recycled Polyamide 6 via Reactive Extrusion: Effect of Chain Extender Types. Polym. Degrad. Stab. 2019, 162, 76–84. 10.1016/j.polymdegradstab.2019.01.026.

[ref67] Wang Y.; Chiao S. M.; Hung T. F.; Yang S. Y. Improvement in Toughness and Heat Resistance of Poly(Lactic Acid)/Polycarbonate Blend through Twin-Screw Blending: Influence of Compatibilizer Type. J. Appl. Polym. Sci. 2012, 125 (S2), E402–E412. 10.1002/app.36920.

[ref68] Bhattacharjee S.; Bajwa D. S. Degradation in the Mechanical and Thermo-Mechanical Properties of Natural Fiber Filled Polymer Composites Due to Recycling. Constr. Build. Mater. 2018, 172, 1–9. 10.1016/j.conbuildmat.2018.03.010.

[ref69] Kodal M. Polypropylene/Polyamide 6/POSS Ternary Nanocomposites: Effects of POSS Nanoparticles on the Compatibility. Polymer 2016, 105, 43–50. 10.1016/j.polymer.2016.10.021.

[ref70] Sharma A. Synthesis and Thermo-Rheological Properties of Thermoplastic Elastomers Based on Hydrogen-Bonded Hard Segments; Catholic University of Louvain Polytechnic School of Louvain: Germany, 2019. http://hdl.handle.net/2078.1/217850 (accessed Jan 17, 2022).

[ref71] Sohel M. A.; Mandal A.; Mondal A.; Pan S.; SenGupta A. Thermal Analysis of ABS/PA6 Polymer Blend Using Differential Scanning Calorimetry. J. Therm. Anal. Calorim. 2017, 129 (3), 1689–1695. 10.1007/s10973-017-6312-6.

[ref72] Zembouai I.; Bruzaud S.; Kaci M.; Benhamida A.; Corre Y. M.; Grohens Y. Mechanical Recycling of Poly(3-Hydroxybutyrate-Co-3-Hydroxyvalerate)/Polylactide Based Blends. J. Polym. Environ. 2014, 22 (4), 449–459. 10.1007/s10924-014-0684-5.

[ref73] Kuram E.; Ozcelik B.; Yilmaz F.; Timur G.; Sahin Z. M. The Effect of Recycling Number on the Mechanical, Chemical, Thermal, and Rheological Properties of PBT/PC/ABS Ternary Blends: With and without Glass-Fiber. Polym. Compos. 2014, 35 (10), 2074–2084. 10.1002/pc.22869.

[ref74] Aurrekoetxea J.; Sarrionandia M. A.; Urrutibeascoa I.; Maspoch M. L. Effects of Recycling on the Microstructure and the Mechanical Properties of Isotactic Polypropylene. J. Mater. Sci. 2001, 36, 2607–2613. 10.1023/A:1017983907260.

[ref75] Rath T.; Kumar S.; Mahaling R. N.; Khatua B. B.; Das C. K.; Yadaw S. B. Mechanical, Morphological and Thermal Properties of in Situ Ternary Composites Based on Poly(Ether Imide), Silicone Rubber and Liquid Crystalline Polymer. Mater. Sci. Eng., A 2008, 490 (1–2), 198–207. 10.1016/j.msea.2008.01.076.

[ref76] Kaci M.; Hamma A.; Pillin I.; Grohens Y. Effect of Reprocessing Cycles on the Morphology and Properties of Poly(Propylene)/Wood Flour Composites Compatibilized with EBAGMA Terpolymer. Macromol. Mater. Eng. 2009, 294 (8), 532–540. 10.1002/mame.200900089.

[ref77] Wu H.; Lv S.; He Y.; Qu J. P. The Study of the Thermomechanical Degradation and Mechanical Properties of PET Recycled by Industrial-Scale Elongational Processing. Polym. Test. 2019, 77, 105882 10.1016/j.polymertesting.2019.04.029.

[ref78] Delva L.; Ragaert K.; Degrieck J.; Cardon L. The Effect of Multiple Extrusions on the Properties of Montmorillonite Filled Polypropylene. Polymers 2014, 6 (12), 2912–2927. 10.3390/polym6122912.

[ref79] Dadras Chomachayi M.; Jalali-arani A.; Martínez Urreaga J. A Comparison of the Effect of Silk Fibroin Nanoparticles and Microfibers on the Reprocessing and Biodegradability of PLA/PCL Blends. J. Polym. Environ. 2021, 29 (8), 2585–2597. 10.1007/s10924-021-02053-1.

[ref80] Song H. J.; Chen X. D.; Fan J. C.; Xu Q. J. Balanced Strength and Toughness Improvement in Polylactide (PLA)/Poly(1,4-Cyclohexylene Dimethylene Terephthalate Glycol) (PCTG) Blends Using Various Compatibilizers. Iran. Polym. J. 2019, 28 (11), 991–999. 10.1007/s13726-019-00763-x.

