# Supplementary material for: Enhanced Recyclability of Thermoplastic Elastomer Toughened Polyamide 6 via Tri- and Multi-epoxy-Terminated POSS Hybrid Additives
Source: ACS Omega. 2024 Oct 30;9(45):45467–86. doi: 10.1021/acsomega.4c07547 (PMC11561595; doi:10.1021/acsomega.4c07547)
Supplement: Supplementary file 1 — ao4c07547_si_001.pdf [file ao4c07547_si_001.pdf]

Supporting Information

Enhanced Recyclability of Thermoplastic Elastomer Toughened Polyamide 6 via Tri- and Multi-Epoxy Terminated POSS Hybrid Additives

Rumeysa Yıldırım[1], Olcay Mert[2,3], Güralp Özkoç[4,5,6], Mehmet Kodal[1,2*]

[1]Chemical Engineering Department, Kocaeli University, 41001, Kocaeli, Türkiye

[2]Polymer Science and Technology Graduate Programme, Kocaeli University, 41001, Kocaeli, Türkiye

[3]Department of Chemistry, Kocaeli University, 41001, Kocaeli, Türkiye

[4]Nanotechnology Research and Application Center SUNUM, Sabanci University, 34956, İstanbul, Türkiye

[5]Department of Chemistry, İstinye University, 34010, İstanbul, Türkiye

[6]Xplore Instruments B.V., 6135 KT, Sittard, The Netherlands

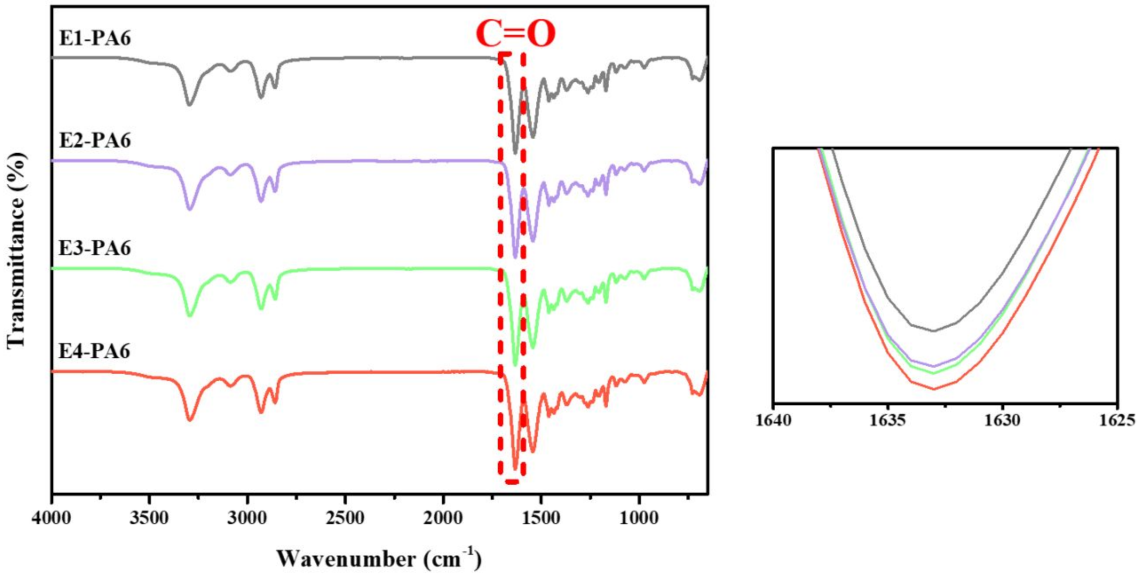

Figure S1. FTIR spectra of PA6 after repeated extrusion cycles

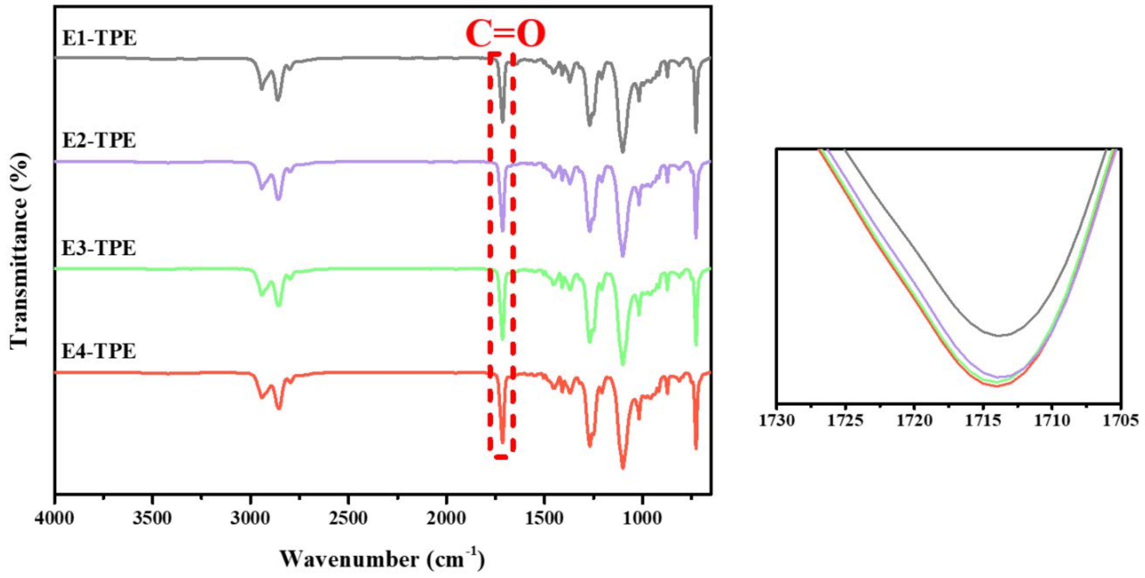

Figure S2. FTIR spectra of TPE after repeated extrusion cycles

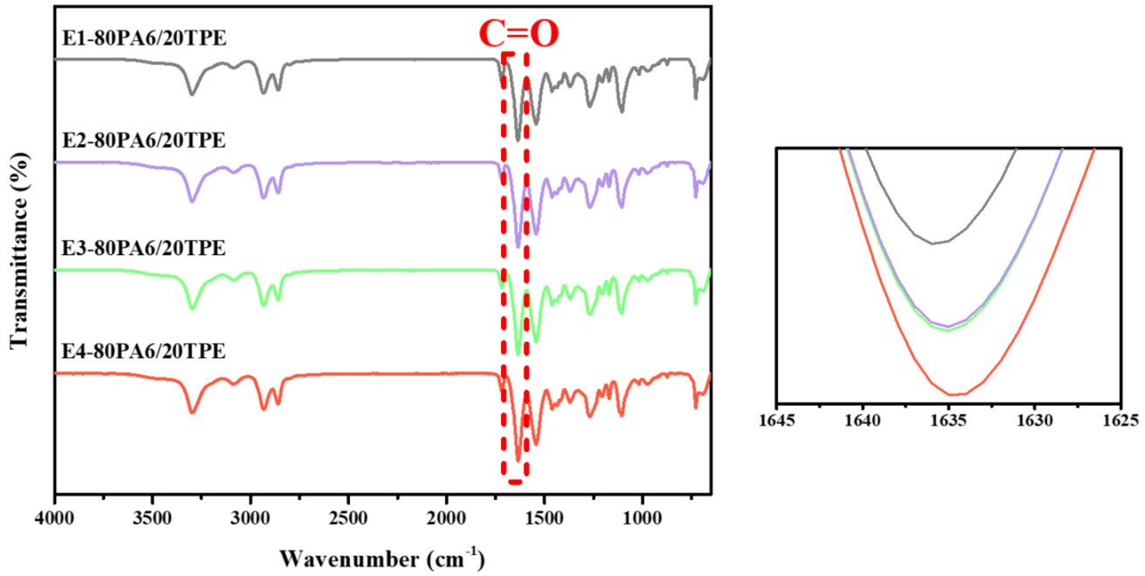

Figure S3. FTIR spectra of 80PA6/20TPE blend after repeated extrusion cycles

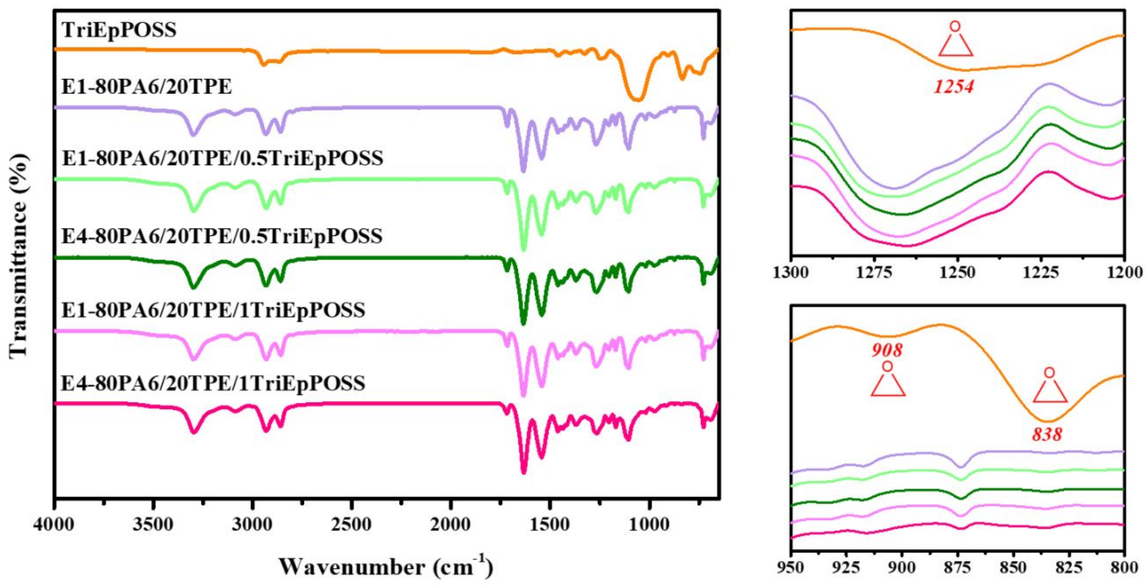

Figure S4. FTIR spectra of TriEpPOSS, E1-80PA6/20TPE, and 80PA6/20TPE/TriEpPOSS blends with respect to POSS loading level after the first and fourth reprocessing cycles

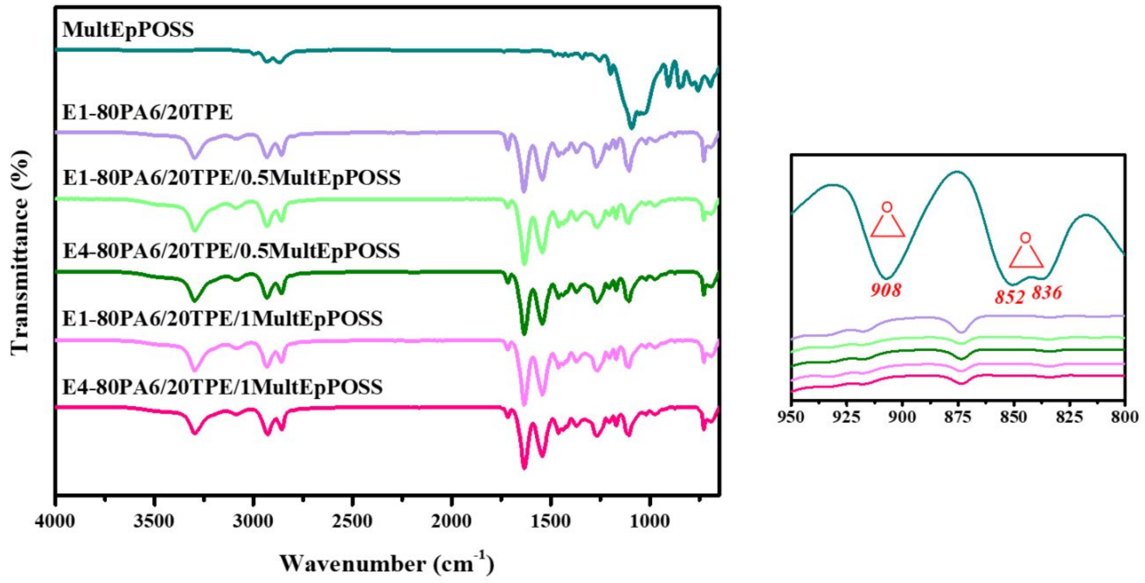

Figure S5. FTIR spectra of MultEpPOSS, E1-80PA6/20TPE and and 80PA6/20TPE/ MultEpPOSS blends with respect to POSS loading level after the first and fourth reprocessing cycles
